# Electroacupuncture ameliorates chronic heart failure: the role of CRH neurons in the paraventricularnucleus of the hypothalamus

**DOI:** 10.3389/fnins.2026.1741523

**Published:** 2026-02-25

**Authors:** Wenyuan Xu, Yujie Guo, Jiaying Wang, Xianghu Zhao, Lian Cai, Zhihao Ren, Shuai Cui, Haosheng Wu, Nenggui Xu, Shengbing Wu, Meiqi Zhou

**Affiliations:** 1 Graduate School, Anhui University of Chinese Medicine, Hefei, China; 2 Department of Rehabilitation Medicine, Zhongda Hospital, Southeast University, Nanjing, China; 3 Institute of Acupuncture and Meridian, Anhui Academy of Chinese Medicine, Hefei, China; 4 Anhui Province Key Laboratory of Meridian Viscera Correlationship, Hefei, China; 5 South China Research Center for Acupuncture and Moxibustion, Medical College of Acu-Moxi and Rehabilitation, Guangzhou University of Chinese Medicine, Guangzhou, China

**Keywords:** chronic heart failure, corticotropin-releasing hormone neurons, electroacupuncture, neural mechanism, paraventricular nucleus of hypothalamus

## Abstract

**Background:**

Chronic heart failure (CHF) constitutes the terminal stage of ischemic heart disease and is characterized by a high mortality rate. Our previous studies have demonstrated that electroacupuncture (EA) modulates the paraventricular nucleus (PVN) of the hypothalamus, thereby exerting a cardioprotective effect against myocardial ischemia. However, the specific mechanisms underlying the role of corticotropin-releasing hormone (CRH) neurons, which are critical for regulating sympathetic outflow and stress responses within the PVN, remain unclear in the context of CHF. Additionally, CHF has been proven to cause an imbalance in the autonomic nervous system. This study seeks to investigate whether EA can re-establish cardiac autonomic homeostasis and ameliorate CHF by modulating PVN^CRH^ neurons, thereby suppressing excessive sympathetic outflow.

**Methods:**

The CHF rat model was established via permanent ligation of the left anterior descending coronary artery. Subsequently, EA was administered at the HT7 (*Shenmen*) acupoint. ECG signals were recorded, and heart rate variability analyzed. The neural connection was demonstrated using viral tracing techniques. Electrophysiological techniques recorded PVN neuron activity, while echocardiography measured left ventricular function. ELISA detected serum N-terminal pro-brain natriuretic peptide and norepinephrine levels. Histological heart examination used staining methods, and c-Fos-positive neurons expression in the PVN was assessed. The PVN was lesioned with kainic acid, and PVN^CRH^ neurons activity was modulated using chemogenetics.

**Results:**

This study indicated that EA significantly ameliorated CHF. In the CHF rat model, we observed excessive activation of the sympathetic nervous system, concomitant with an increased number of c-Fos-positive neurons in the PVN. EA intervention effectively reversed these changes. Viral tracing revealed neural connections between the heart, HT7 acupoint, and PVN. Following lesioning of the PVN, the therapeutic efficacy of EA was attenuated. Furthermore, chemogenetic experiments revealed that inhibiting the activity of PVN^CRH^ neurons produced a protective effect against CHF similar to that of EA, whereas activating these neurons counteracted the protective effects of EA.

**Conclusion:**

EA ameliorated CHF by inhibiting the activity of PVN^CRH^, suppressing sympathetic overactivation. These findings provided scientific evidence for the potential clinical application of acupuncture in cardiovascular disease management.

## 1 Background

Ischemic heart disease (IHD) remains the leading cause of death worldwide, claiming approximately 9 million lives annually and placing a significant burden on global healthcare systems (Chong et al., 2024). Acute myocardial infarction, one of the most severe manifestations of IHD, affects over 7 million people annually, with nearly one-third developing post-infarction chronic heart failure (CHF) within 5 years, further exacerbating morbidity and mortality rates (Ran et al., 2025). Notwithstanding the advancements in contemporary pharmacotherapy—exemplified by angiotensin receptor–neprilysin inhibitors and SGLT2 inhibitors—alongside device-based therapeutics, approximately 30% of CHF patients continue to exhibit a suboptimal clinical prognosis (Yang et al., 2024). Acupuncture, as a typical non-pharmacological therapy supported by evidence-based medicine in complementary and alternative medicine, has been systematically recognized by the WHO and medical communities in many countries for its clinical efficacy and safety (Liu et al., 2021). Over the past few years, numerous researches, including our researches, have shown that acupuncture can regulate the central autonomic nervous system through integrated effects involving multi-pathway, multi-level, and multi-target, thereby improving cardiovascular function (Wang S. et al., 2024; Fan et al., 2020; Cui et al., 2018). This provides scientific evidence for the mechanism of acupuncture for cardiovascular diseases (CVDs) from the perspective of neurobrain science.

Cardiac function is governed by the reciprocal regulation of the sympathetic and parasympathetic nervous systems, which maintain physiological homeostasis of heart rate, rhythm, and myocardial contractility through a dynamic autonomic equilibrium (Goldberger et al., 2019; Zipes, 1990). Under pathological conditions, the heart exhibits significant autonomic dysregulation, characterized by prolonged sympathetic hyperactivation and concurrent vagal withdrawal. This sympathovagal imbalance serves as a critical pathophysiological driver in the initiation and progression of CVDs (Manolis et al., 2021; He et al., 2016). To investigate these central mechanisms, rodent models of CHF have been widely utilized, particularly those induced by coronary artery ligation, as they effectively recapitulate the sustained sympathetic overdrive observed in clinical patients (Pfeffer et al., 1979; Mark, 1995).

The hypothalamic paraventricular nucleus (PVN) serves as a pivotal integration hub within the brain, exerting significant control over neuroendocrine functions and autonomic cardiovascular modulation (Li and Pan, 2007; Dampney et al., 2018; Pyner, 2014). Parvocellular neurons within the PVN send monosynaptic projections to autonomic centers in the brainstem and the intermediolateral cell column of the spinal cord. This circuitry constitutes the primary descending pathway for sympathetic regulation, exerting direct control over chronotropic, inotropic, and vasomotor functions (Nunn et al., 2011). In addition, CRH neurons play a central role in coordinating neuroendocrine responses to stress and regulating autonomic nervous system output (Jiang et al., 2018). Although CRH neurons are distributed across multiple brain regions, they exhibit particularly dense distribution and functional dominance in the PVN. Heightened expression or pathological secretion of CRH within the PVN triggers sustained hyperactivation of both the hypothalamic–pituitary–adrenal axis and the sympathetic nervous system. This neuroendocrine dysregulation represents a pivotal mechanism in the aberrant central control of cardiovascular function (Herman et al., 2016). Notably, various diseases, including hypertension, myocardial ischemia, arrhythmias, and especially CHF, are closely associated with abnormal sympathetic innervation and enhanced sympathetic nervous system activity (Triposkiadis et al., 2009; Chang and Ramchandra, 2025).

Electroacupuncture (EA), as a modern modification of traditional acupuncture, offers clinical advantages such as proven efficacy, quantifiable parameters, and convenient operation. Although existing evidence suggests that EA has significant therapeutic effects on CVDs, its specific mechanisms remain unclear (Shao et al., 2025; Wang et al., 2022). In preclinical disease models, numerous studies have revealed that EA can inhibit PVN neural activity and modulate neurotransmitters to reduce sympathetic tone (Zhou et al., 2024). Our previous research (Kun et al., 2023; Wu et al., 2025) has confirmed that the PVN is a key central target for EA in alleviating myocardial ischemia. Based on our preliminary findings, this study hypothesizes that EA therapy may exert its effects by modulating CRH neurons within the PVN of the hypothalamus. This action is proposed to inhibit excessive sympathetic nervous system activation, thereby rebalancing cardiac autonomic function and ameliorating congestive heart failure.

## 2 Methods

### 2.1 Animals

The experimental animals used in this study were clean-grade Sprague–Dawley male rats, 8 weeks old, weighing 200–250 g, and purchased from Henan Skebes Biotechnology Co., Ltd. [Production Licence Number: SCXK (YU) 2020–0005]. These rats were housed in the experimental animal facility of the Anhui Province Key Laboratory of Meridian Viscera Correlationship, where the environmental conditions were maintained at a temperature of 24 ± 2 °C, relative humidity of 50–60%, and a 12 h light–dark cycle. During the housing period, the animals had free access to food and water. All experiments were conducted after the rats had undergone a 1-week adaptation period. Our work adhered to “the Guide for the Care and Use of Laboratory Animals” issued by the Chinese National Institutes of Health and was approved by the Animal Care and Use Committee of Anhui University of Chinese Medicine, with approval number AHUCM-rats-2024145.

### 2.2 Animal grouping

Our work comprises four independent experiments, and the detailed grouping information is summarized in Supplementary Table S1. In experiment I, 24 rats were randomly divided into the following three groups: (1) the Sham group (*n* = 6), rats underwent sham CHF model surgery only. (2) The CHF group (*n* = 6), rats underwent CHF modeling surgery only. (3) The EA group (*n* = 6), rats were subjected to EA intervention following the establishment of the CHF model. (4) the Sham EA group (*n* = 6), rats were subjected to sham EA intervention following the establishment of the CHF model. Additionally, to verify the neural connections between the heart, the *Shenmen* (HT7) acupoint, and the PVN, the tracer virus was injected into the heart (*n* = 3) and the acupoint (*n* = 3). To ensure the precise location of the PVN, cholera toxin subunit B (CTB) was injected into the PVN for tracing (*n* = 3).

In experiment II, we performed lesioning of the PVN region, and 24 rats were randomly assigned to the following three groups: (1) the CHF + Saline group (*n* = 6), saline was injected into the PVN of the CHF rats. (2) The CHF + Saline+EA group (*n* = 6), following the injection of saline into the PVN of the CHF rats, EA intervention was performed. (3) The CHF + KA group (*n* = 6), kainic acid (KA) was injected into the PVN of the CHF rats. (4) The CHF + KA + EA group (*n* = 6), following the injection of KA into the PVN of the CHF rats, EA intervention was performed.

In Experiment III, we modulated CRH neurons in the PVN using the inhibitory virus, and 24 rats were randomly assigned to the following four groups: (1) The Sham+mCherry+CNO group (*n* = 6): The PVN of sham rats was injected with the control virus (rAAV-EF1α-DIO-mCherry, expressing only fluorescent protein). Following viral expression, clozapine-N-oxide (CNO) was intraperitoneally injected. This group served as a vector control to exclude off-target effects. (2) The CHF + mCherry+CNO group (*n* = 6): The PVN of CHF rats was injected with the control virus. Following viral expression, CNO was intraperitoneally injected. (3) The CHF + hM4Di + CNO group (*n* = 6): The PVN of CHF rats was injected with the inhibitory virus (rAAV-EF1α-DIO-hM4Di-mCherry). Following viral expression, CNO was intraperitoneally injected to activate the virus and inhibit neuronal activity. (4) The CHF + hM4Di + Saline group (*n* = 6): The PVN of CHF rats was injected with the inhibitory virus. Following viral expression, saline was intraperitoneally injected.

In Experiment IV, CRH neurons in the PVN were modulated using the excitatory virus, and EA intervention was performed. 24 rats were randomly divided into the following four groups: (1) The CHF + mCherry+CNO group (*n* = 6): Similar to Experiment III, CHF rats received the control virus followed by CNO injection. (2) The CHF + mCherry+CNO + EA group (*n* = 6): The control virus was injected into the PVN of CHF rats. Following viral expression, CNO was administered via intraperitoneal injection and combined with EA intervention. (3) The CHF + hM3Dq + CNO + EA group (*n* = 6): The excitatory virus (rAAV-EF1α-DIO-hM3Dq-mCherry) was injected into the PVN of CHF rats. Following viral expression, CNO was intraperitoneally injected to activate the virus and excite neuronal activity during EA intervention. (4) The CHF + hM3Dq + Saline+EA group (*n* = 6): The excitatory virus was injected into the PVN of CHF rats. Following viral expression, saline was administered via intraperitoneal injection combined with EA intervention.

The sample size was determined based on the “Resource Equation Method” to strictly adhere to animal welfare ethics (Reduction) while ensuring statistical validity (Charan and Kantharia, 2013). The study comprised four independent experimental series. For four experiments (4 groups each, *n* = 6), *E* was 20. These values fall within the optimal range (10–20) recommended for animal research. This sample size strategy is also consistent with established protocols in previous studies on EA for cardiovascular diseases (Wu et al., 2025; Zhou et al., 2025). Furthermore, post-hoc power analyses indicated that the statistical power (1-*β*) for key outcome measures exceeded 0.90 across all major comparisons, confirming the adequacy of the sample size.

### 2.3 Chronic heart failure model

**Figure 1:**
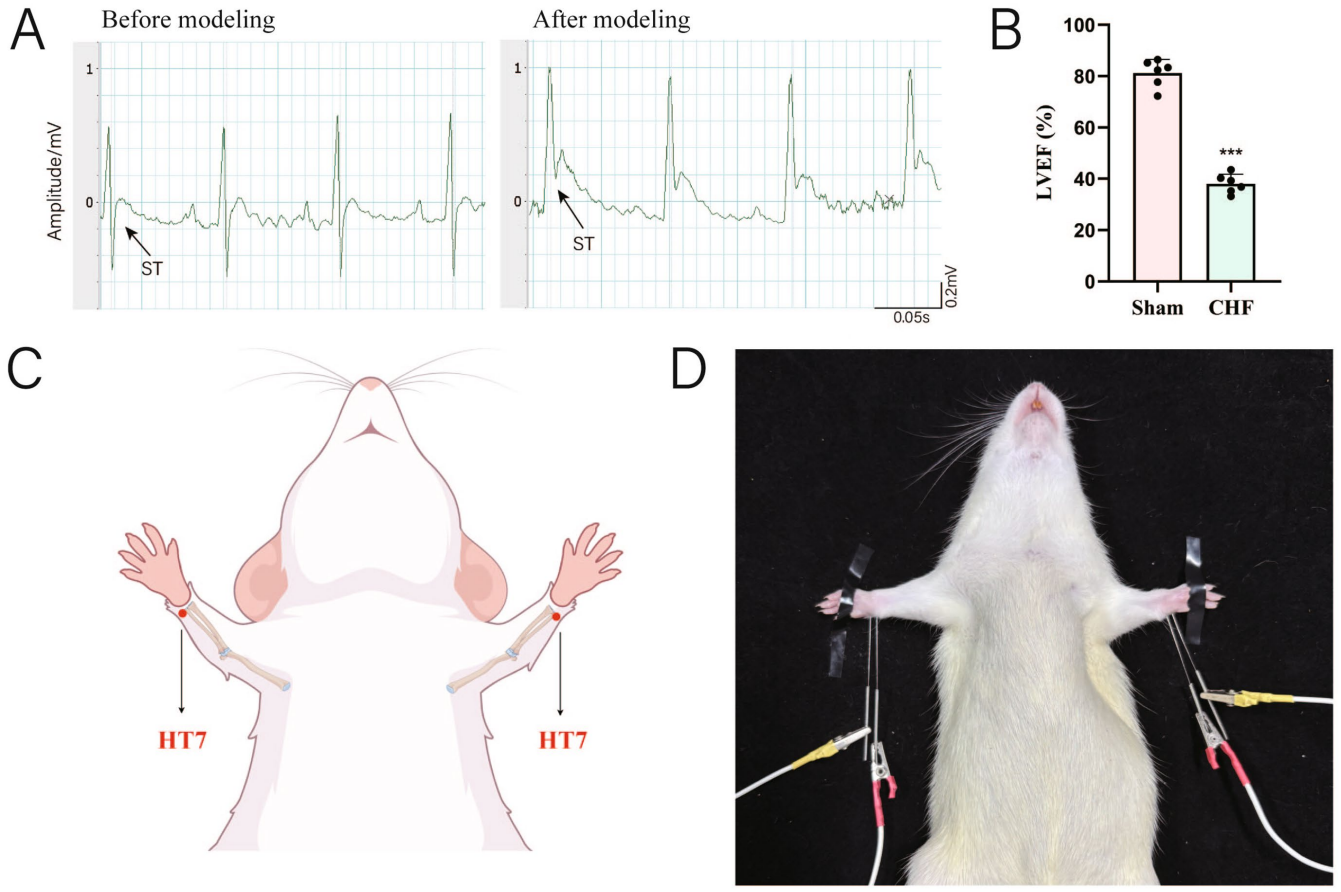
Schematic diagram of CHF modeling ECG and EA intervention. **(A)** Changes in the ST segment of the ECG before and after ligation of the LAD. **(B)** The statistical analysis of LVEF values following 4 weeks of LAD ligation (biological replicates *n* = 3, mean ± SD). ^***^p < 0.001 vs. the sham group. **(C)** Photograph of the rat undergoing EA at the HT7 acupoint (vertical insertion, 2 Hz, 1 mA, depth 2–3 mm). **(D)** Anatomical localization of the HT7 acupoint.

The CHF rat model was established using the left anterior descending (LAD) artery ligation method (Litwin et al., 1994). Rats were fasted for 12 h prior to surgery but allowed access to water. General anesthesia was induced using 3% isoflurane (R510-22-10, RWD Life Science Co., Ltd.), and once unconscious, the rats were secured to the operating table. Anesthesia was maintained with 1.5–2% isoflurane. During anesthesia, standard lead electrocardiograms (ECG) were recorded using the Powerlab Standard Limb II lead system (ML118, AD Instruments International Trading Co., Ltd.). For surgical procedures, the hair on the left anterior chest was shaved and the area disinfected. A vertical incision was made on the left anterior chest perpendicular to the ribs, and the pectoralis major and minor muscles were bluntly dissected. A curved hemostatic forceps was inserted into the thoracic cavity at the fourth intercostal space, and the intercostal space was dilated using a chest expander to extrude the heart. The LAD branch of the coronary artery was ligated using 7–0 non-absorbable suture material at a position 2–3 mm below the left atrial appendage and a needle insertion depth of 0.5 mm to induce ischemia. After ligation, quickly reposition the heart into the thoracic cavity and expel any air from the thoracic cavity. ST-segment elevation (≥0.2 mV) on the ECG is used as the criterion for successful ligation (Figure 1A). Subsequently, layered sutures were performed using 4–0 absorbable sutures, and rats were given intraperitoneal injections of penicillin (200,000 U/mL, 0.5 mL/d) for three consecutive days to prevent infection. Four weeks (28 days) postoperatively, left ventricular ejection fraction (LVEF) was assessed via echocardiography. The model was considered successful if the LVEF value was ≤ 45% (Yuan et al., 2018; Figure 1B). The sham group underwent a single needle puncture at the corresponding site after thoracotomy without ligating the LAD coronary artery. Postoperatively, rats were monitored until recovery from anesthesia and administered penicillin via intraperitoneal injection to prevent infection and Carprofen (5 mg/kg, s.c.) to alleviate pain and distress daily for three consecutive days. Rats with abnormal ECG preoperatively, those that died during the experimental period, and those with unsuccessful modeling were excluded from the study. To maintain a consistent sample size, any excluded animals were replaced by additional rats that underwent the same procedures and met the inclusion criteria.

### 2.4 Electroacupuncture intervention

Based on published techniques for locating acupoints in experimental animals and comparisons with primate models (Shu et al., 2024), our study selected the *Shenmen* (HT7) point on the Hand-Shaoyin Heart Meridian for EA intervention. This point is situated at the wrist, on the palmar side of the transverse wrist crease, at the ulnar end, within the radial notch of the flexor carpi ulnaris tendon (Figure 1C). Prior to EA intervention, the bilateral *Shenmen* point regions in rats were disinfected according to standard protocol. Disposable sterile acupuncture needles (0.25 × 25 mm, Suzhou Tianxie Acupuncture Instrument Co., Ltd.) were inserted perpendicularly into the point to a depth of 2–3 mm. Following insertion, the HT7 point was connected to the negative electrode of the EA device (HANS-200A, Nanjing Jisheng Medical Technology Co., Ltd.), while the area 1 mm lateral to the point was connected to the positive electrode. The intervention employed a continuous current wave with a frequency of 2 Hz and an amperage of 1 mA, with stimulation intensity calibrated to elicit slight limb tremors (Figure 1D).

For the Sham EA group, a minimal acupuncture protocol widely adopted in previous studies was employed to strictly control for non-specific effects such as cutaneous stimulation (Wan et al., 2025; Xu et al., 2026; Guo et al., 2026). In this group, sterile needles were inserted shallowly into the subcutaneous tissue to a depth of 0.5 to 1 mm at the same acupoint without connecting to the electrical stimulator or delivering any current.

All EA and sham EA interventions were performed under isoflurane-induced anesthesia, administered once daily for 30 min over seven consecutive days. The control group, not receiving EA treatment, underwent anesthesia for the same duration.

### 2.5 Stereotaxic injection

Under isoflurane anesthesia (induction at 3%, maintenance at 1%), rats were secured in a stereotaxic apparatus. The ear bars were symmetrically positioned to ensure that the skull was horizontally aligned. The adequacy of fixation was verified by the alignment of the nose, the absence of head movement, and the skull’s horizontal orientation. After the scalp was shaved and disinfected, a midline incision was performed, and the dura mater was removed to expose Bregma. A burr hole was drilled at the coordinates corresponding to the PVN (relative to Bregma: anteroposterior [AP] ± 1.92 mm, mediolateral [ML] ± 0.35 mm, dorsoventral [DV] ± 7.6 mm), as per the stereotaxic coordinates from a rat brain atlas.

In accordance with the experimental grouping described previously, a microinjector (R480, RWD Life Science Co., Ltd.) was used to inject 30 nL of the following solutions into the PVN at a rate of 10 nL/min: CTB (CTB-555, Wuhan Shumi Brain Science and Technology Co., Ltd.), KA (K0250, Shanghai Sigma Audley Trading Co., Ltd.), saline (ST341, Beyotime Biotechnology Co., Ltd.), inhibitory virus [the 1:1 mixture of rAAV-CRH-CRE-WPRE-hGH polyA and rAAV-EF1α-DIO-hM4D(Gi)-mCherry-WPREs, Wuhan Shumi Brain Science and Technology Co., Ltd.], excitatory virus [the 1:1 mixture of rAAV-CRH-CRE-WPRE-hGH polyA and rAAV-EF1α-DIO-hM3D(Gq)-mCherry-WPREs, Wuhan Shumi Brain Science and Technology Co., Ltd.], and control virus (rAAV-EF1α-DIO-mCherry-WPRE-hGH, Wuhan Shumi Brain Science and Technology Co., Ltd.). After injection, the microinjection needle was left in place for 10 min, and the incision was closed with sterile sutures. Postoperatively, rats were monitored until recovery from anesthesia and administered penicillin via intraperitoneal injection to prevent infection and Carprofen (5 mg/kg, s.c.) to alleviate pain and distress daily for three consecutive days. After data collection from each rat, histological identification was performed by perfusion and sectioning to observe the injection site and confirm its accuracy, and data from inaccurate sites were excluded.

### 2.6 Microinjection

We employed pseudorabies virus (PRV) and herpes simplex virus (HSV) as trans-synaptic tracers to map the multi-synaptic neural circuits connecting the periphery to the central nervous system. To verify the neural connection between the heart and the PVN, we performed a microinjection of a tracing virus into the rat heart. Rats were anesthetized (induction at 3%, maintenance at 1%) and secured on an animal operating table, followed by tracheal intubation and connection to the small animal ventilator (R415, RWD Life Science Co., Ltd.). Once the physiological state of the rat stabilized, the chest was prepared and disinfected. A thoracotomy was then performed, and the sternum was retracted using a rib spreader to expose the heart. A microinjection of PRV tracing virus (PRV-CAG-mRFP, Wuhan Shumi Brain Science and Technology Co., Ltd.) was administered into the left ventricular wall at three distinct sites, with a volume of 1 μL per site and a rate of 100 nL/s. After the injection, the thoracic incision was sutured and disinfected.

To verify the neural connection between the HT7 acupoint and the PVN, we performed a microinjection of a tracing virus into the HT7 acupoint region. Rats were anesthetized (induction at 3%, maintenance at 1%) and secured on an animal operating table, and the area surrounding the HT7 acupoint was depilated, prepared, and disinfected. The skin was then incised to facilitate needle insertion. A microinjection of HSV tracing virus (HSV-EGFP, Wuhan Shumi Brain Science and Technology Co., Ltd.) was administered at three distinct sites 2–3 mm beneath the skin of the acupoint, with a volume of 1 μL per site and a rate of 100 nL/min. After the injection, the incision was sutured and disinfected. Postoperatively, rats were monitored until recovery from anesthesia and administered penicillin via intraperitoneal injection to prevent infection and Carprofen (5 mg/kg, s.c.) to alleviate pain and distress daily for three consecutive days.

Five days after the microinjection of the tracing virus, when the rats exhibited significant abnormal conditions, they were euthanized by continuous anesthesia with 5% isoflurane. The brain tissue was then extracted and observed under the microscope (magnification: × 10; DP72, Olympus Optical Co., Ltd.) to examine the viral tracing in the PVN region.

### 2.7 Electrocardiogram and heart rate variability analysis

The ECG was recorded from rats using the Powerlab Standard Limb II lead system (ML118, AD Instruments International Trading Co., Ltd.). Rats were anesthetised (induction at 3%, maintenance at 1%) and secured to an animal restraint table. Needle electrodes were inserted subcutaneously into the right forelimb and left hindlimb, and intramuscularly into the right hindlimb. ECG recording commenced once stable waveforms were obtained. Each animal was recorded for 30 min. Following completion, electrodes were removed, rat skin cleaned, and equipment switched off. Heart rate variability (HRV) was measured using the HRV module in LabChart 8 software. Quantitative analysis was performed in the low-frequency (LF, 0.2–0.75 Hz), high-frequency (HF, 0.75–2.5 Hz), and LF/HF ratio of the power spectrum.

### 2.8 Echocardiography

Rats were anesthetized with isoflurane (induction at 3%, maintenance at 1%) and secured in a supine position on the animal platform. Chest hair was removed using a depilatory agent, and a coupling agent was applied. Cardiac function was then assessed using a digital ultrasound system (VINNO6 Lab, Feiyino Technology Co., Ltd.) equipped with an 18 MHz echo transducer. Then used M-mode recording to measure LVEF and left ventricular short-axis shortening (LVFS). For each rat, three technical replicate echocardiographic measurements were performed, and the mean value was calculated.

### 2.9 Electrophysiology

Neuronal discharge in the PVN brain region of rats was recorded using electrophysiological techniques. Rats were anesthetized (induction at 3%, maintenance at 1%) and secured in a stereotaxic apparatus using the same method as in the brain stereotaxic surgery. The scalp hair was shaved, disinfected, and a midline incision was made to remove the dura mater and expose Bregma. As described previously, a cranial hole was drilled above the PVN brain region after locating it. Subsequently, an eight-channel (2 × 4) microelectrode array (designed, Kewa Suzhou Medical Technology Co., Ltd.) was implanted. The electrode was slowly advanced to the target brain region at a rate of 5 μm/s using electric propulsion. Once stable neural activity was observed, recording began and lasted for 400 s. After recording, the incision was sutured with sterile sutures, and the rats were given intraperitoneal injections of penicillin for three consecutive days to prevent infection. The OmniPlex multichannel acquisition system (version 1.20.0, Beijing Plexon Technology Co., Ltd.) was used to record neuronal discharge (filtered at 150–8000 Hz, sampling rate 40 kHz) and field potentials (filtered at 0.7–400 Hz, sampling rate 1 kHz). After data acquisition, Offline Sorter software (version 4.7.2, Beijing Plexon Technology Co., Ltd.) was first used to remove typical high-amplitude interference signals using the waveform cross-correlation method. Subsequently, NeuroExplorer software (version 5, Beijing Plexon Technology Co., Ltd.) was employed to analyze neuronal signals. To strictly define functional activity and exclude background noise, neurons were classified as “active” only if they exhibited a spontaneous mean firing rate greater than 2 Hz and a stable signal-to-noise ratio exceeding 3:1 throughout the recording period (Quiroga et al., 2004; Luther and Tasker, 2000; Buzsáki, 2004). Based on these inclusion criteria, spike discharge raster diagrams, autocorrelograms, and spectrograms were generated to compare the frequency and characteristics of neuronal signals among the groups. Postoperatively, rats were monitored until recovery from anesthesia and administered penicillin via intraperitoneal injection to prevent infection and Carprofen (5 mg/kg, s.c.) to alleviate pain and distress daily for three consecutive days. Finally, histological identification was performed. After tissue fixation and sectioning, the location was observed to confirm the accuracy of the electrode implantation site.

### 2.10 Animal euthanasia and tissue collection

All experiments in this work were conducted in accordance with the principle of minimizing animal suffering. After the intervention, rats were subjected to tissue collection. Blood was first collected via intraperitoneal puncture, followed by the extraction of the heart and brain tissues. This sequence prevented blood loss and coagulation due to the removal of the heart and brain, thereby ensuring the quality and quantity of the blood samples. Prior to blood collection, rats were anesthetized (induction at 3%, maintenance at 1%), and blood was drawn from the abdominal aorta. Subsequently, rats were euthanized by continuous induction with 5% isoflurane until cessation of heartbeat and respiration, confirming death. The heart and brain tissues were then harvested. Brain tissues were immediately rinsed in phosphate buffered saline (PBS) to remove surface blood and post-fixed by immersion in 4% paraformaldehyde at 4 °C for 24–48 h. Throughout the blood collection and tissue extraction procedures, strict aseptic techniques were adhered to in order to prevent sample contamination. The samples were stored according to experimental requirements for subsequent analyses.

### 2.11 Enzyme-linked immunosorbent assay

After blood collection from the abdominal aorta of rats, the blood was placed vertically in a refrigerator at 4 °C and allowed to coagulate naturally for 10–20 min. Subsequently, the blood was centrifuged in a high-speed refrigerated centrifuge (4 °C, 3000 revolutions per min, 15 min). The supernatant obtained from centrifugation was prepared as standard samples. Standard samples were prepared, spiked, and incubated in strict accordance with the manufacturer’s instructions for the Enzyme-linked immunosorbent assay (ELISA) kit (AF03205-B, AF03182-B, Hunan Aifang Biotechnology Co., Ltd.). Absorbance values were measured using an ELISA instruction, and the serum levels of N-terminal pro-brain natriuretic peptide (NT-proBNP) and norepinephrine (NE) in each sample group were calculated based on the standard curve generated.

### 2.12 HE staining

After blood collection, the diaphragm and pericardium were incised, and the heart was carefully extracted using curved forceps. The heart was then rinsed in pre-cooled 0.9% sodium chloride solution at 4 °C. A transverse incision was made 5 mm above the apex of the heart. Hematoxylin–eosin (HE) staining is employed to assess pathological changes in myocardial tissue. The procedure for HE staining is as follows: Initially, paraffin-embedded tissue sections are placed on a staining rack and subjected to deparaffinization by sequential immersion in xylene I, II, and III for 3 min each, followed by rehydration through a graded series of ethanol solutions (absolute ethanol I for 2 min, absolute ethanol II for 2 min, 95% ethanol for 1 min, 90% ethanol for 1 min, and 80% ethanol for 1 min). After rehydration, the sections are briefly drained on a staining rack for 30 s. The sections are then stained with hematoxylin for 10 min, followed by a gentle rinse in tap water to remove excess stain. Subsequently, the sections are differentiated in a 1% hydrochloric acid-ethanol solution for 6 s, drained for 4 s, and then soaked in tap water for 10 min. For counterstaining, the sections are immersed in eosin for 2.5 min, followed by a 30 s drain on the staining rack. Dehydration is performed through a graded ethanol series (80% ethanol for 10 s, 90% ethanol for 10 s, 95% ethanol for 2 min, absolute ethanol I for 3 min, absolute ethanol II for 3 min, and absolute ethanol III for 3 min). The sections are then cleared in xylene (xylene I, II, and III for 3 min each). Finally, the sections are mounted with neutral resin, taking care to avoid the formation of air bubbles, and allowed to dry at room temperature for 2–3 d before examination under a microscope (magnification: × 20; DP72, Olympus Optical Co., Ltd.).

### 2.13 Masson staining

Masson staining was employed to evaluate the condition of myocardial fibrosis. The procedure was executed with precision using a Masson staining kit, as described herein. Initially, tissue sections were placed on a staining rack and subjected to deparaffinization by sequential immersion in xylene I, II, and III for 3 min each, followed by a rehydration series through graded ethanol solutions (95, 70, and 30%) and distilled water for 2 min each. Subsequently, the sections underwent a warm water rinse at 30–40 °C for 30–60 s twice, ensuring the removal of all droplets except for the samples. The staining process involved hematoxylin staining for 60 s, followed by a tap water rinse for 30 s, and then Masson’s trichrome staining for 30–60 s with another tap water rinse. Differentiation was performed in a 6–8% phosphoric acid solution for 6–8 min, adjusted based on the appearance of collagen fibers under a microscope (magnification: × 20; DP72, Olympus Optical Co., Ltd.) until they turned pink. Counterstaining with light green was applied for 5 min, followed by a final tap water rinse. Dehydration was achieved through a series of ethanol solutions and xylene, culminating in mounting with neutral resin. To quantify collagen deposition within the myocardial tissue, the collagen volume fraction (CVF) was determined using ImageJ software (version 6.0).

### 2.14 Nissl staining

To verify the accuracy of the stereotaxic injection and the extent of the excitotoxic lesions, brain sections containing the PVN were processed for Nissl staining. Briefly, after the brain tissues were harvested and fixed in 4% paraformaldehyde as described above, they were dehydrated and sectioned at a thickness of 30 μm. The sections were mounted on gelatin-coated slides, air-dried, and rehydrated. They were then stained with 0.1% cresyl violet solution (Sigma-Aldrich, St. Louis, MO, United States) for 10–15 min at room temperature. Following staining, the sections were differentiated in 95% ethanol, dehydrated through a graded series of alcohols (70, 90, and 100%), cleared in xylene, and cover slipped with neutral gum. The stained sections were examined under a light microscope (Olympus, Tokyo, Japan) to assess neuronal survival. Lesioned areas were identified by the loss of Nissl bodies, neuronal shrinkage (pyknosis), and the absence of normal neuronal cytoarchitecture compared to the intact surrounding regions.

### 2.15 Immunofluorescence staining

Fixed brain tissues were dehydrated in graded sucrose solutions (15 and 30%) and sectioned into 30-μm coronal slices using a cryostat. The rat brain slices were immersed in PBS and washed three times, each for 5 min. Subsequently, the brain slices were placed in a blocking buffer solution containing 0.5% Triton X-100 and 3% bovine serum albumin (BSA) for 1 h at room temperature.

For c-Fos expression analysis, the slices were incubated with the primary antibody (2250S, Saixintong Shanghai Biological Reagents Co., Ltd.) at a 1:500 dilution overnight at 4 °C. For the co-localization analysis of virus and CRH neurons, the slices were incubated with anti-CRH antibody (10944-1-AP, Wuhan Sanying Biotechnology Co., Ltd.) overnight at 4 °C. On the following day, the slices were rinsed three times with PBS, each for 5 min. After rinsing, the slices were immersed in a buffer solution containing 0.5% Triton X-100 and 3% BSA. Corresponding secondary antibodies (111–545-003, Shanghai Youningwei Biotechnology Co., Ltd.) were added at a 1:500 dilution, and the mixture was incubated at room temperature in the dark for 2 h. Following this, the slices were rinsed three times with PBS, mounted with an anti-fluorescence quenching solution containing 4′,6-diamidino-2-phenylindole (DAPI, abs9235-25 mL, Aibixin Shanghai Biotechnology Co., Ltd.), and stained. After staining, three images were randomly selected for photography. Images were captured using a panoramic tissue section imaging system (magnification: × 20; PanoBrain, Meca Scientific, PB-ST100, Shanghai BOYE Biotechnology Co., Ltd.). The number of c-Fos-positive neurons was analyzed using ImageJ software (version 6.0). For co-localization verification, the endogenous fluorescence of mCherry and the immunofluorescence of CRH were observed, and the percentage of mCherry-positive neurons co-expressing CRH was calculated.

### 2.16 Statistical analysis

All data were analyzed using GraphPad Prism (version 8.0) and presented as the mean ± standard deviation (S. D.). Normality was assessed via the Shapiro–Wilk test, while homogeneity of variance was evaluated using the Brown-Forsythe test. For comparisons between two groups, data were initially tested for normality. Those satisfying normality criteria were analyzed using either the Student’s T-test or Paired T-test. Data that failed to meet normality assumptions were subjected to the Mann–Whitney U test or Wilcoxon signed-rank test. For comparisons among multiple groups, one-way analysis of variance (ANOVA) was employed if normality and homogeneity assumptions were met, followed by the Holm–Šidák (or Bonferroni) *post-hoc* test to correct for multiple comparisons. If assumptions were not met, the Kruskal-Wallis H test was used, followed by Dunn’s post hoc test. Correlations were assessed using Pearson’s correlation coefficient. To control for the inflated type I error rate due to multiple testing in correlation analyses, *p*-values were adjusted using the Benjamini-Hochberg False Discovery Rate (FDR) method (or Bonferroni correction). Statistical significance was set at *p* < 0.05.

## 3 Results

### 3.1 Effects of EA at HT7 on cardiac function in rats with CHF

**Figure 2:**
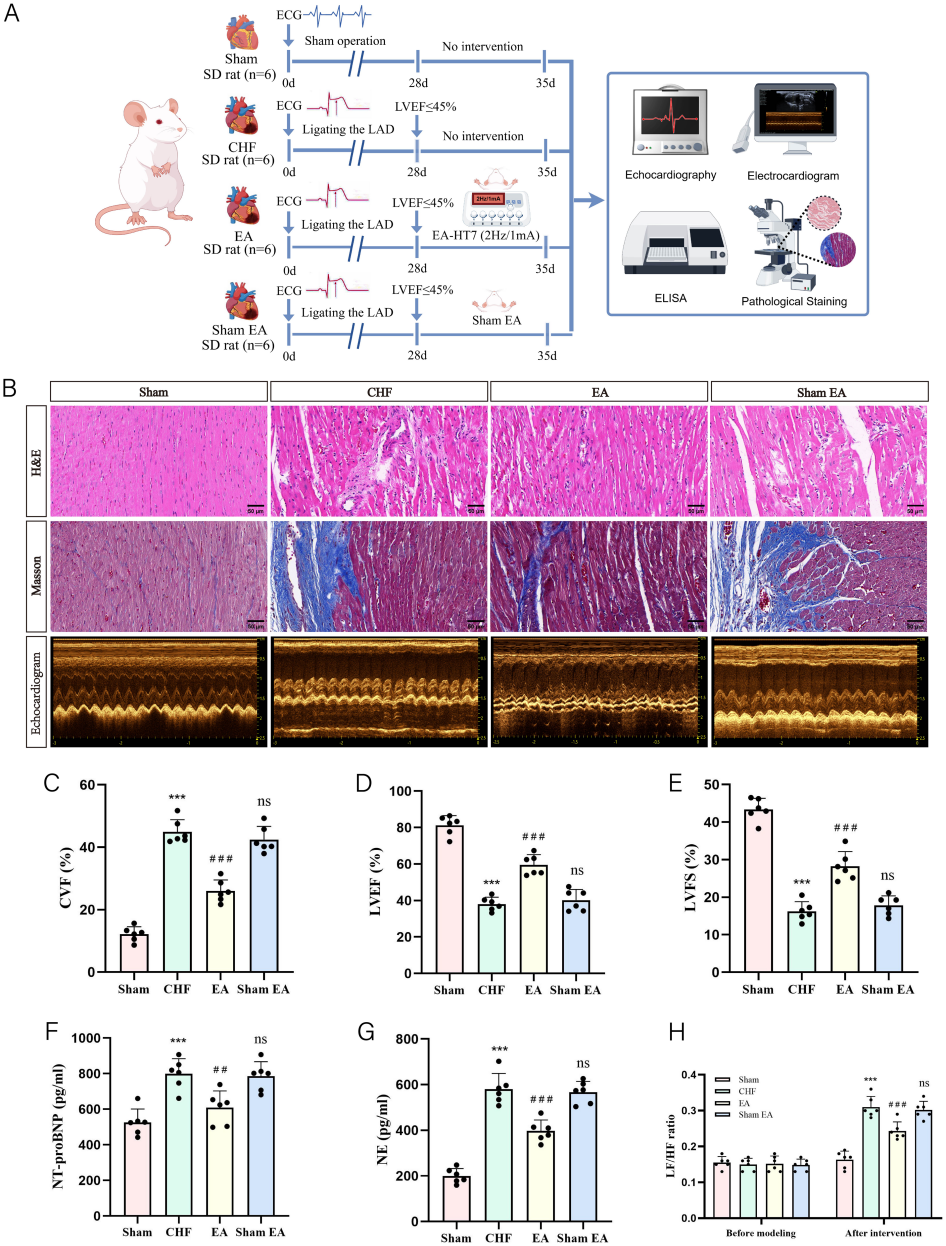
The effect of EA on cardiac function in CHF rats. **(A)** Experiment timeline. The experimental design involved three groups of rats: Sham, CHF, EA, and Sham EA. Sham rats (*n* = 6) underwent a sham operation and were monitored for 35 days without intervention. CHF rats (*n* = 6) had the LAD ligated, leading to CHF, with no treatment until day 35. EA rats (*n* = 6) received LAD ligation followed by EA treatment (2 Hz/1 mA at HT7) from day 28 to 35. Sham EA rats (*n* = 6) received LAD ligation followed by sham EA treatment (superficial insertion, no current) from day 28 to 35. Outcomes were assessed via echocardiography, electrocardiography, ELISA, and pathological staining. **(B)** Representative images of HE staining, Masson staining, and echocardiographic images (scale bar = 50 μm). **(C)** The statistical analysis of CVF values (biological replicates *n* = 6, mean ± SD). ^***^p < 0.001 vs. the sham group; ^###^*p* < 0.001 vs. the CHF group; ns, not significant vs. the CHF group. **(D)** The statistical analysis of LVEF values (biological replicates *n* = 6, mean ± SD). ^***^p < 0.001 vs. the sham group; ^###^*p* < 0.001 vs. the CHF group; ns, not significant vs. the CHF group. **(E)** The statistical analysis of LVFS values (biological replicates *n* = 6, mean ± SD). ^***^p < 0.001 vs. the sham group; ^###^*p* < 0.001 vs. the CHF group; ns, not significant vs. the CHF group. **(F)** The statistical analysis of NT-proBNP values (biological replicates *n* = 6, mean ± SD). ^***^p < 0.001 vs. the sham group; ^##^*p* < 0.001 vs. the CHF group; ns, not significant vs. the CHF group. **(G)** The statistical analysis of NE values (biological replicates *n* = 6, mean ± SD). ^***^p < 0.001 vs. the sham group; ^###^*p* < 0.001 vs. the CHF group; ns, not significant vs. the CHF group. **(H)** The statistical analysis of LF/HF ratio (biological replicates *n* = 6, mean ± SD). ^***^p < 0.001 vs. the sham group; ^###^*p* < 0.001 vs. the CHF group; ns, not significant vs. the CHF group.

To assess the effects of EA on CHF, the model was established via LAD ligation followed by EA or Sham EA treatment. Systematic evaluations including histology, echocardiography, and biochemical assays were conducted (Figure 2A).

Histological analysis showed that the CHF group exhibited significant cardiomyocyte edema, inflammation, and severe fibrosis. EA treatment markedly improved myocardial structure and reduced fibrosis, whereas the Sham EA group retained severe pathological changes and high CVF levels comparable to the CHF group (Figures 2B,C).

Functionally, EA treatment significantly reversed the reduced LVEF and LVFS observed in CHF rats while the Sham EA group failed to improve cardiac function (Figures 2D,E). Consistently, EA lowered the elevated serum NT-proBNP, NE levels, and HRV LF/HF ratio which suggested reduced cardiac burden and restored autonomic balance. In contrast, the Sham EA group showed no significant improvement in these biochemical and autonomic parameters compared to the CHF group (Figures 2F–H).

Overall, EA exerted significant cardioprotective effects. Given the lack of therapeutic efficacy in the Sham EA group, we reasoned that the underlying central neural mechanisms would remain consistent with the CHF baseline. Therefore, the Sham EA group was excluded from subsequent invasive mechanistic analyses to focus on the specific mechanisms of effective EA treatment.

### 3.2 PVN participation in EA at HT7 treatment for rats with CHF

**Figure 3:**
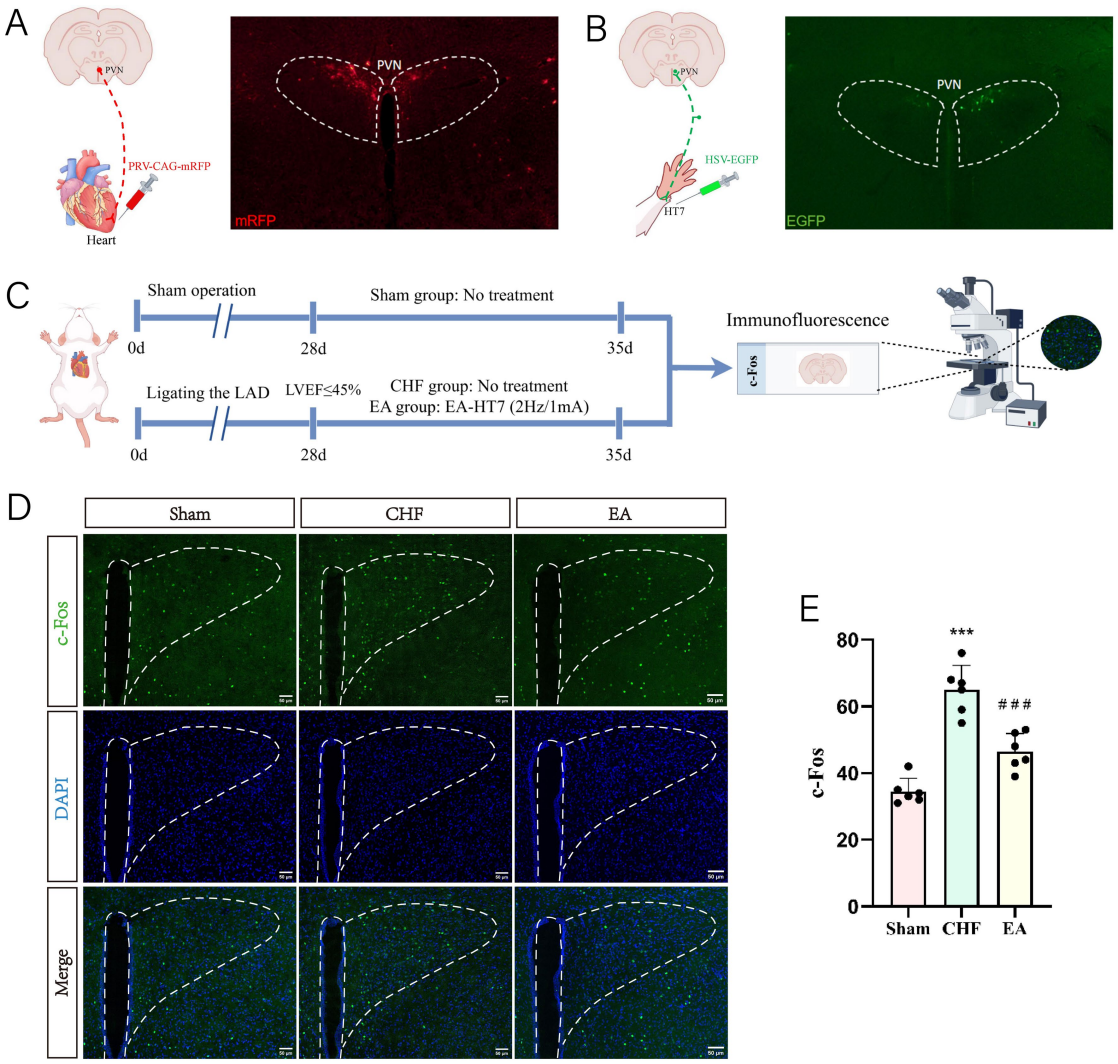
Neural connections between the heart, HT7 acupoint, and the PVN, and c-Fos expression in the PVN across different groups. **(A)** Neural connections between the heart and the PVN (biological replicates *n* = 3). **(B)** Neural connections between the HT7 and the PVN (biological replicates *n* = 3). **(C)** Experiment timeline. The experiment included three groups: Sham, CHF, and EA. At day 0, sham rats underwent a sham operation, CHF rats underwent LAD ligation, and EA rats received LAD ligation followed by EA treatment from day 28 to 35. On day 35, immunofluorescence assessments of the PVN were conducted for all groups. **(D)** Representative images of c-Fos expression (biological replicates *n* = 3, scale bar = 50 μm). **(E)** The statistical analysis of c-Fos expression in the PVN (biological replicates *n* = 6, mean ± SD). ^***^p < 0.001 vs. the sham group; ^###^*p* < 0.001 vs. the CHF group.

Following the administration of PRV, a transneuronal tracer known for its ability to map motor efferent pathways, into the heart and HSV, a tool used for tracing sensory afferent pathways, into the HT7 acupoint, we observed corresponding viral fluorescence in the PVN of the rat brain. The presence of PRV in the PVN indicates a motor efferent pathway from the PVN to the heart, suggesting that the PVN can modulate cardiac function (Figure 3A). Conversely, the detection of HSV in the PVN signifies the sensory afferent pathway from the HT7 acupoint, indicating that sensory information from HT7 is transmitted to the PVN (Figure 3B). Together, these findings confirm the existence of neural circuits connecting the heart, HT7 acupoint, and PVN, thereby establishing a functional link between acupuncture stimulation at HT7 and cardiac regulation via the PVN. These viral tracing results qualitatively confirm the structural existence of the neural circuit connecting the heart, PVN, and HT7. The specific functional involvement of CRH neurons within this circuit was subsequently investigated using cell-type specific chemogenetics in the following experiments.

Immunofluorescence staining was used to detect c-Fos-positive neurons in the PVN (Figure 3C). Results showed significant upregulation in the CHF group compared with the sham group, which was downregulated by EA (Figures 3D,E). This indicated that EA inhibits the expression of c-Fos in the PVN.

### 3.3 Effects of EA at HT7 on the electrophysiological activity of the PVN

**Figure 4:**
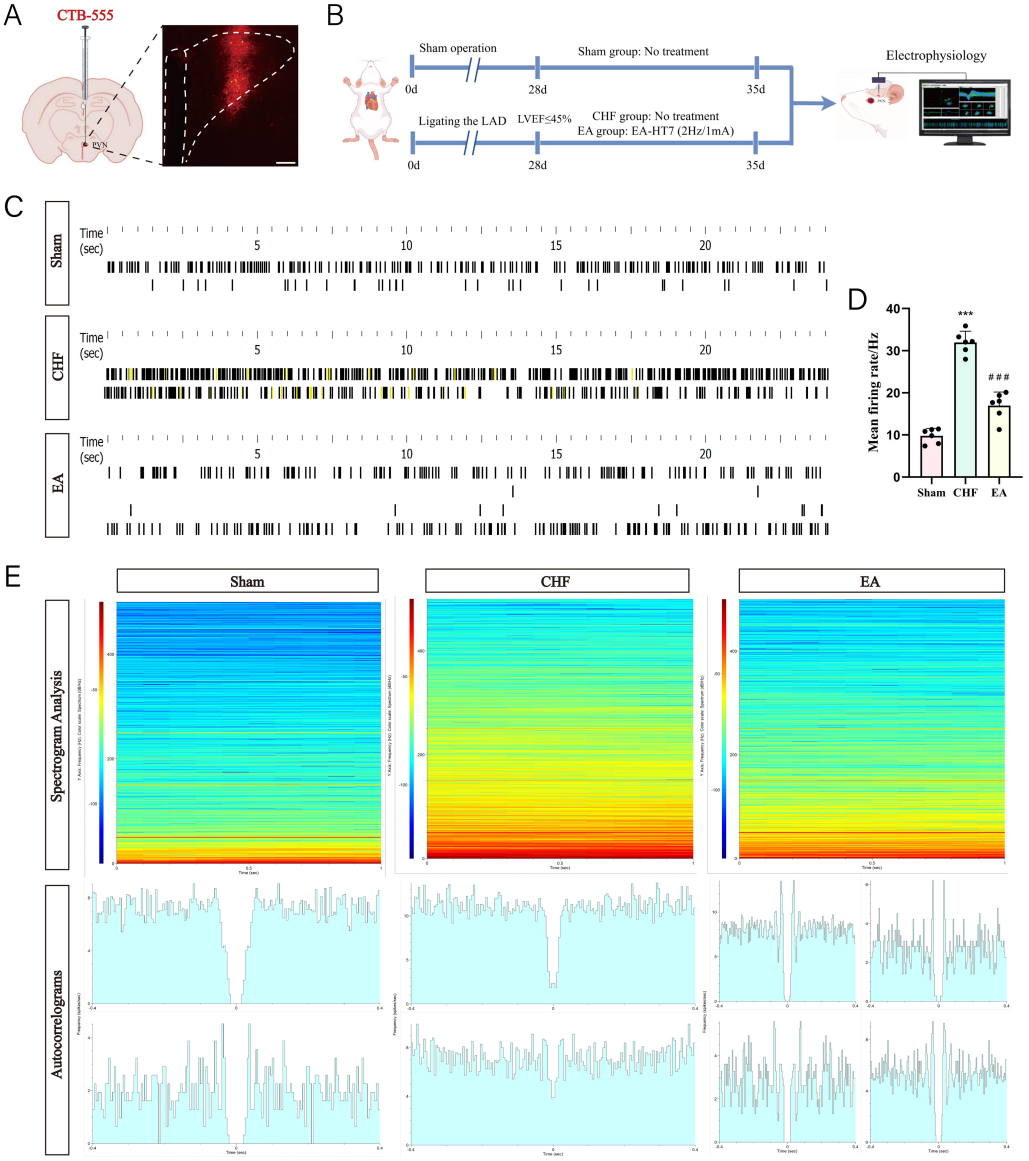
Effects of EA at HT7 on the electrophysiological activity of the PVN. **(A)** Injection site diagram (biological replicates *n* = 3, scale bar = 50 μm). **(B)** Experiment timeline. The experiment included three groups: Sham, CHF, and EA. At day 0, Sham rats underwent a sham operation, CHF rats underwent LAD ligation, and EA rats received LAD ligation followed by EA treatment from day 28 to 35. Electrophysiological assessments of the PVN were conducted on day 35. **(C)** Spike discharge raster diagram of neurons. **(D)** The statistical analysis of the PVN neuronal activity frequency (biological replicates *n* = 3, mean ± SD). ^***^p < 0.001 vs. the sham group; ^###^*p* < 0.001 vs. the CHF group. **(E)** Spectrograms analysis and autocorrelation analysis.

The location of the PVN was confirmed by administering the CTB injection (Figure 4A). Electrophysiological recordings showed that PVN neuronal activity frequency was higher in the CHF group than in the sham group and lower in the EA group than in the CHF group (Figures 4B–D). Spectrograms analysis revealed higher spectral energy in the CHF group than in the sham group and lower in the EA group than in the CHF group (Figure 4E). Autocorrelation analysis indicated two active neurons in the sham and CHF groups, with four neurons showing enhanced activity after EA at the *Shenmen* acupoint (Figure 4E).

### 3.4 Correlation between PVN neuronal activity, autonomic balance, and cardiac function

**Figure 5:**
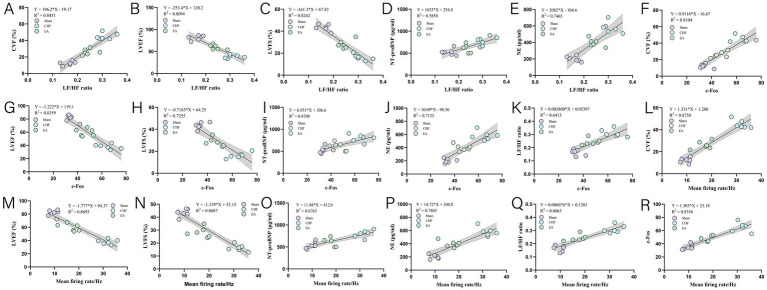
Correlation between PVN neuronal activity, autonomic balance, and cardiac function. Pearson correlation analyses were performed to examine the linear relationships between variables. Data for each scatter plot were pooled from the Sham, CHF, and EA groups (*n* = 6 rats per group), resulting in a total sample size of *n* = 18 for each correlation. The solid lines represent the linear regression fits. **(A)** *Y* = 196.2*X–19.17, *R*^2^ = 0.8451. **(B)** *Y* = −253.4*X + 120.2, *R*^2^ = 0.8094. **(C)** *Y* = −161.3*X + 67.83, *R*^2^ = 0.8242. **(D)** *Y* = 1633*X + 254.8, *R*^2^ = 0.5850. **(E)** *Y* = 2082*X–104.6, *R*^2^ = 0.7465. **(F)** *Y* = 0.9116*X–16.67, *R*^2^ = 0.8104. **(G)** *Y* = −1.222*X + 119.1, *R*^2^ = 0.8359. **(H)** *Y* = −0.7183*X + 64.25, *R*^2^ = 0.7255. **(I)** *Y* = 6.951*X + 306.6, *R*^2^ = 0.4708. **(J)** *Y* = 10.09*X–98.56, *R*^2^ = 0.7135. **(K)** *Y* = 0.003800*X + 0.05397, *R*^2^ = 0.6413. **(L)** *Y* = 1.351*X + 1.280, *R*^2^ = 0.8750. **(M)** *Y* = −1.777*X + 94.37, *R*^2^ = 0.8693. **(N)** *Y* = −1.129*X + 52.15, *R*^2^ = 0.8687. **(O)** *Y* = 11.88*X + 412.6, *R*^2^ = 0.6763. **(P)** *Y* = 14.72*X + 104.8, *R*^2^ = 0.7465. **(Q)** *Y* = 0.006076*X + 0.1201, *R*^2^ = 0.8063. **(R)** *Y* = 1.303*X + 23.18, *R*^2^ = 0.8356.

To elucidate the underlying mechanisms, we performed a series of correlation analyses. The LF/HF ratio was positively correlated with CVF, NT-proBNP, and NE, and negatively correlated with LVEF and LVFS (Figures 5A–E). The expression of c-Fos was positively associated with CVF, NT-proBNP, NE, and the LF/HF ratio, and negatively associated with LVEF and LVFS (Figures 5F–K). The mean firing rate was positively correlated with CVF, NT-proBNP, NE, the LF/HF ratio, and c-Fos expression, and negatively correlated with LVEF and LVFS (Figures 5L-R).

Collectively, these findings delineate a strong statistical association among the variables: in CHF, PVN neuronal activation (marked by c-Fos) is positively correlated with autonomic imbalance (elevated LF/HF ratio) and deteriorated cardiac function. This suggests that the therapeutic effects of EA may be linked to its modulation of this axis.

### 3.5 Effects of EA at HT7 on cardiac function in rats with CHF after PVN lesion

**Figure 6:**
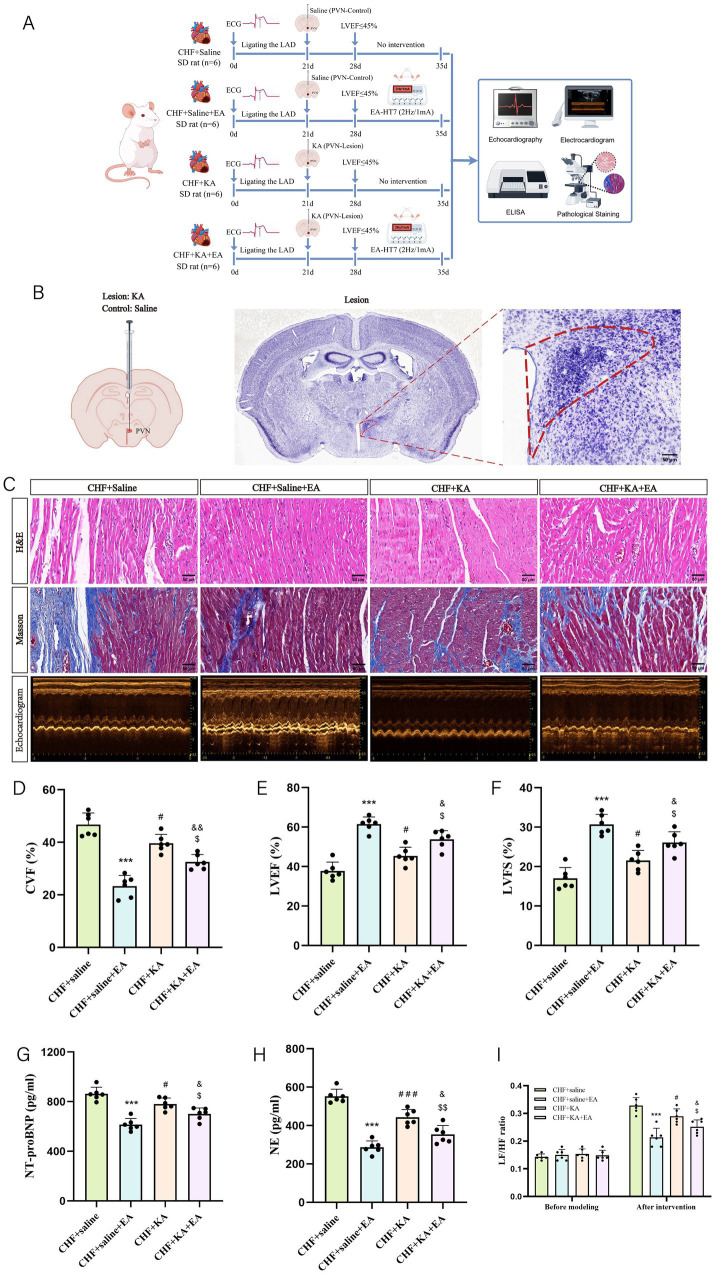
The effect of EA on cardiac function in rats with CHF after PVN lesion. **(A)** Experiment timeline. The experimental design involved rats subjected to LAD ligation on day 0. The CHF + Saline group received saline injections into the PVN on day 21 and no further intervention until day 35. The CHF + Saline + EA group underwent EA treatment (2 Hz/1 mA at HT7) from day 28 to 35. The CHF + KA group received KA to lesion the PVN on day 21 and no further intervention until day 35. The CHF + KA + EA group received KA to lesion the PVN on day 21, followed by EA treatment similarly from day 28 to 35. Assessments included echocardiography, electrocardiography, ELISA, and pathological staining. **(B)** Microinjection diagram and Nissl staining of the PVN region. **(C)** Representative images of HE staining, Masson staining, and echocardiographic images (scale bar = 50 μm). **(D)** The statistical analysis of CVF values (biological replicates *n* = 6, mean ± SD). ^***^p < 0.001 vs. the CHF + saline group; ^#^*p* < 0.05 vs. the CHF + Saline group; ^$^*p* < 0.05 vs. the CHF + KA group; ^&&^*p* < 0.01 vs. the CHF + Saline+EA group. **(E)** The statistical analysis of LVEF values (biological replicates *n* = 6, mean ± SD). ^***^p < 0.001 vs. the CHF + saline group; ^#^*p* < 0.05 vs. the CHF + Saline group; ^$^*p* < 0.05 vs. the CHF + KA group; ^&^*p* < 0.05 vs. the CHF + Saline+EA group. **(F)** The statistical analysis of LVFS values (biological replicates *n* = 6, mean ± SD). ^***^p < 0.001 vs. the CHF + saline group; ^#^*p* < 0.05 vs. the CHF + Saline group; ^$^*p* < 0.05 vs. the CHF + KA group; ^&^*p* < 0.05 vs. the CHF + Saline+EA group. **(G)** The statistical analysis of NT-proBNP values (biological replicates *n* = 6, mean ± SD). ^***^p < 0.001 vs. the CHF + saline group; ^#^*p* < 0.05 vs. the CHF + Saline group; ^$^*p* < 0.05 vs. the CHF + KA group; ^&^*p* < 0.05 vs. the CHF + Saline+EA group. **(H)** The statistical analysis of NE values (biological replicates *n* = 6, mean ± SD). ^***^p < 0.001 vs. the CHF + saline group; ^#*##*^*p* < 0.001 vs. the CHF + Saline group; ^$$^*p* < 0.051 vs. the CHF + KA group; ^&^*p* < 0.05 vs. the CHF + Saline+EA group. **(I)** The statistical analysis of LF/HF ratio (biological replicates *n* = 6, mean ± SD). ^***^p < 0.001 vs. the CHF + saline group; ^#^*p* < 0.05 vs. the CHF + Saline group; ^$^*p* < 0.05 vs. the CHF + KA group; ^&^*p* < 0.05 vs. the CHF + Saline+EA group.

To explore the role of the PVN in the effects of EA on CHF, we induced PVN lesions with KA and compared outcomes among the CHF + Saline, CHF + Saline+EA, CHF + KA, and CHF + KA + EA groups (Figures 6A,B). Prior to functional assessment, the accuracy and specificity of the lesions were confirmed via Nissl staining. The PVN region in KA-treated rats exhibited significant neuronal loss, nuclear shrinkage, and necrosis, whereas the surrounding brain tissues remained structurally intact (Figure 6B).

Regarding the impact of the lesion itself, the CHF + KA group exhibited reduced sympathetic activity and slightly improved cardiac function compared to the CHF + Saline group, indicating that ablating the hyperactive PVN offers partial cardioprotection. However, the CHF + Saline+EA group demonstrated the most significant improvements in myocardial structure, reduced fibrosis, and enhanced cardiac function (increased LVEF and LVFS) compared to the CHF + Saline group.

Notably, when EA was applied to lesioned rats, the CHF + KA + EA group showed further improvements compared to the CHF + KA group, suggesting EA exerts residual effects via alternative pathways. Nevertheless, compared to the potent effects in the CHF + Saline+EA group, the CHF + KA + EA group exhibited significantly attenuated cardioprotection, characterized by lower LVEF and LVFS, and higher NT-proBNP and NE levels (Figures 6C–H). HRV analysis confirmed these findings, showing the lowest LF/HF ratio in the CHF + Saline+EA group, whereas the CHF + KA + EA group showed a significantly higher ratio (Figure 6I). These findings underscore the critical role of the PVN in mediating the maximal cardioprotective effects of EA in CHF.

### 3.6 Effects of chemical inhibition of PVN^CRH^ neuron activity on cardiac function in rats with CHF

**Figure 7:**
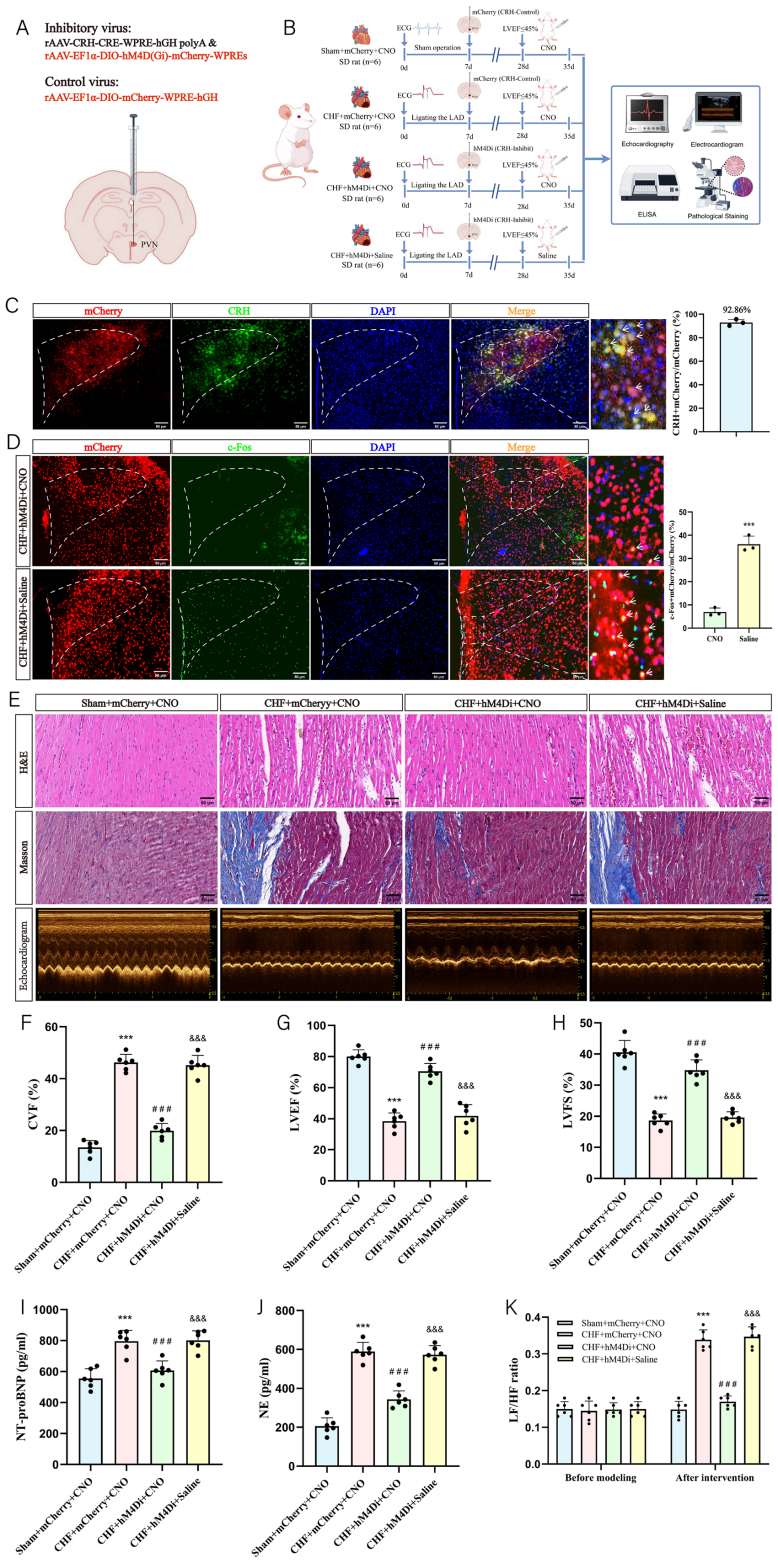
The effect of EA on cardiac function in CHF rats following chemogenetic inhibition of PVN^CRH^ neurons. **(A)** Microinjection diagram. **(B)** Experiment timeline. The experimental design involved four groups: Sham + mCherry + CNO, CHF + mCherry + CNO, CHF + hM4Di + CNO, and CHF + hM4Di + Saline (*n* = 6 per group). On day 0, all groups except the Sham underwent LAD ligation to induce CHF. On day 7, mCherry or hM3Dq was injected into the PVN of the rats in the respective groups. From day 28 to 35, the Sham + mCherry + CNO, CHF + mCherry + CNO, and hM4Di + CNO groups received intraperitoneal injections of CNO, while the CHF + hM4Di + Saline group received saline. Outcomes were assessed using echocardiography, electrocardiography, ELISA, and pathological staining at day 35. **(C)** Verification of viral targeting specificity in the PVN (biological replicates *n* = 3, mean ± SD, scale bar = 50 μm). **(D)** Verification of the functional efficacy of the chemogenetic approach in the PVN (biological replicates *n* = 3, mean ± SD, scale bar = 50 μm). ^***^p < 0.001 vs. CNO. **(E)** Representative images of HE staining, Masson staining, and echocardiographic images (scale bar = 50 μm). **(F)** The statistical analysis of CVF values (biological replicates *n* = 6, mean ± SD). ^***^p < 0.001 vs. the Sham+mCherry+CNO group; ^###^*p* < 0.001 vs. the CHF + mCherry+CNO group; ^&&&^*p* < 0.001 vs. the CHF + hM4Di + CNO group. **(G)** The statistical analysis of LVEF values (biological replicates *n* = 6, mean ± SD). ^***^p < 0.001 vs. the Sham+mCherry+CNO group; ^###^*p* < 0.001 vs. the CHF + mCherry+CNO group; ^&&&^*p* < 0.001 vs. the CHF + hM4Di + CNO group. **(H)** The statistical analysis of LVFS values (biological replicates *n* = 6, mean ± SD). ^***^p < 0.001 vs. the Sham+mCherry+CNO group; ^###^*p* < 0.001 vs. the CHF + mCherry+CNO group; ^&&&^*p* < 0.001 vs. the CHF + hM4Di + CNO group. **(I)** The statistical analysis of NT-proBNP values (biological replicates *n* = 6, mean ± SD). ^***^p < 0.001 vs. the Sham+mCherry+CNO group; ^###^*p* < 0.001 vs. the CHF + mCherry+CNO group; ^&&&^*p* < 0.001 vs. the CHF + hM4Di + CNO group. **(J)** The statistical analysis of NE values (biological replicates *n* = 6, mean ± SD). ^***^p < 0.001 vs. the Sham+mCherry+CNO group; ^###^*p* < 0.001 vs. the CHF + mCherry+CNO group; ^&&&^*p* < 0.001 vs. the CHF + hM4Di + CNO group. **(K)** The statistical analysis of LF/HF ratio (biological replicates *n* = 6, mean ± SD). ^***^p < 0.001 vs. the Sham+mCherry+CNO group; ^###^*p* < 0.001 vs. the CHF + mCherry+CNO group; ^&&&^*p* < 0.001 vs. the CHF + hM4Di + CNO group.

To elucidate the role of CRH neurons in the beneficial effects of EA on CHF, we injected adeno-associated virus (AAV) vectors into the PVN and modulated the activity of CRH neurons using the chemogenetic tool CNO (Figures 7A,B). First, we rigorously validated the specificity and efficacy of our chemogenetic system. Specifically, in the CHF + hM4Di + Saline group, immunofluorescence staining revealed that 92.86% of mCherry-expressing neurons co-localized with endogenous CRH, confirming that the virus specifically targeted CRH neurons with minimal off-target infection (Figure 7C). Subsequently, to verify the functional efficacy of inhibition *in vivo*, we analyzed c-Fos expression. As shown in Figure 7D, CNO administration significantly decreased the percentage of mCherry+ neurons co-expressing c-Fos in the CHF + hM4Di + CNO group compared to the CHF + hM4Di + Saline group, confirming the effective suppression of neuronal activity. Following this validation, we assessed the cardiac outcomes. The CHF + mCherry+CNO group showed severe myocardial damage and increased fibrosis, while the CHF + hM4Di + CNO group exhibited improved structure and reduced fibrosis (Figures 7E,F). Echocardiography revealed reduced LVEF and LVFS in the CHF + mCherry+CNO group, which improved with hM4Di + CNO treatment (Figures 7G,H). Serum NE and NT-proBNP levels decreased with hM4Di + CNO, indicating reduced sympathetic activity (Figures 7I,J). HRV analysis showed improved autonomic balance in the CHF + hM4Di + CNO group (Figure 7K). These findings suggest that inhibiting CRH neurons can ameliorate CHF, similar to EA effects.

### 3.7 Effects of chemical activation of PVN^CRH^ neuron activity on cardiac function in rats with CHF

**Figure 8:**
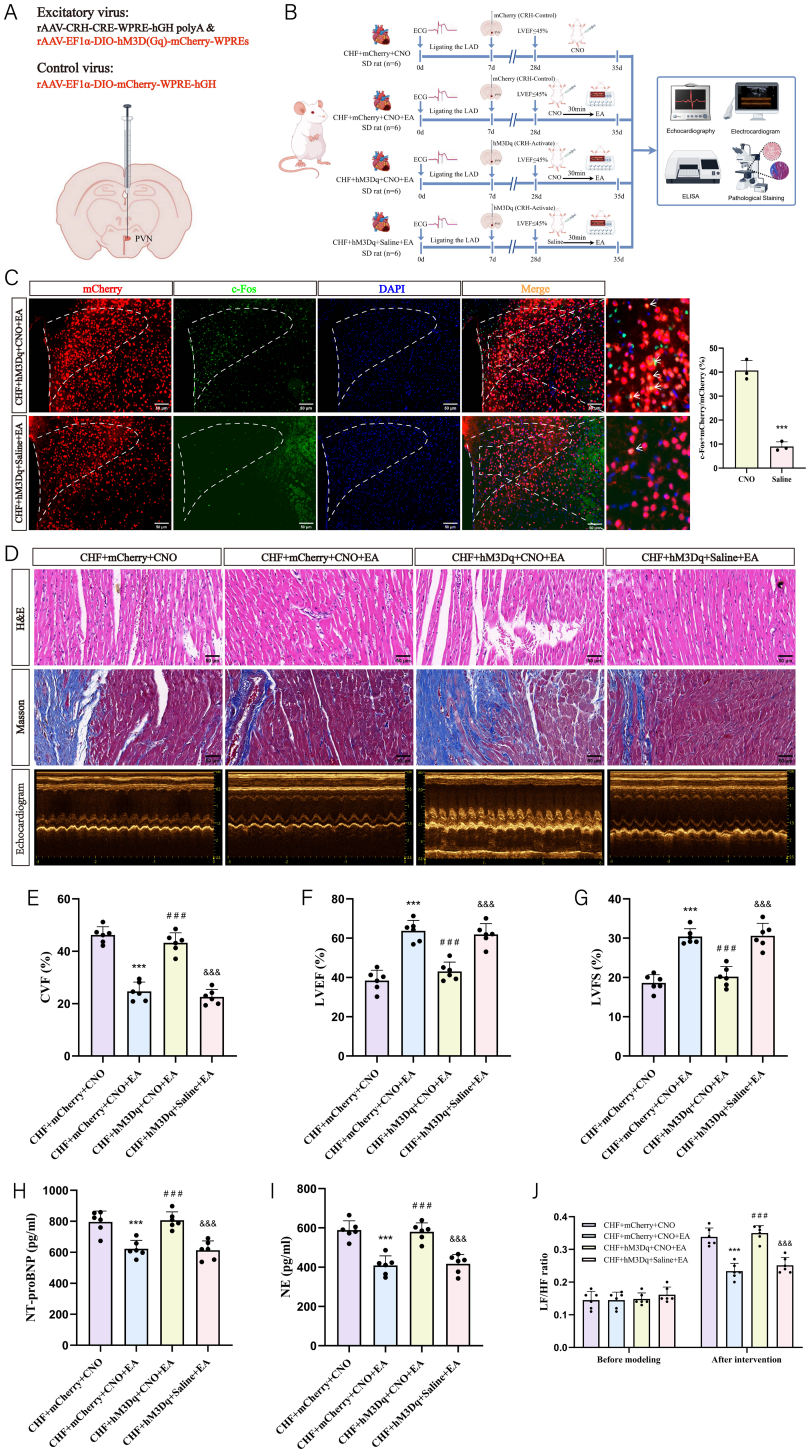
The effect of EA on cardiac function in CHF rats following chemogenetic activation of PVN^CRH^ neurons. **(A)** Microinjection diagram. **(B)** Experiment timeline. The experimental design involved four groups: CHF + mCherry + CNO, CHF + mCherry + CNO + EA, CHF + hM3Dq + CNO + EA, and CHF + hM3Dq + Saline + EA (*n* = 6 per group). LAD ligation was performed on day 0. On day 7, mCherry or hM3Dq was injected into the PVN of the rats in the respective groups. From day 28 to 35, the CHF + mCherry + CNO + EA and CHF + hM3Dq + CNO + EA group received intraperitoneal injections of CNO, and after 30 min, EA treatment was applied. The CHF + hM3Dq + Saline + EA group received saline and underwent EA treatment similarly. Assessments were conducted using echocardiography, electrocardiography, ELISA, and pathological staining. **(C)** Verification of the functional efficacy of the chemogenetic approach in the PVN (biological replicates *n* = 3, mean ± SD, scale bar = 50 μm). ^***^p < 0.001 vs. CNO. **(D)** Representative images of HE staining, Masson staining, and echocardiographic images (scale bar = 50 μm). **(E)** The statistical analysis of CVF values (biological replicates *n* = 6, mean ± SD). ^***^p < 0.001 vs. the CHF + mCherry+CNO group; ^###^*p* < 0.001 vs. the CHF + mCherry+CNO + EA group; ^&&&^*p* < 0.001 vs. the CHF + hM3Dq + CNO + EA group. **(F)** The statistical analysis of LVEF values (biological replicates *n* = 6, mean ± SD). ^***^p < 0.001 vs. the CHF + mCherry+CNO group; ^###^*p* < 0.001 vs. the CHF + mCherry+CNO + EA group; ^&&&^*p* < 0.001 vs. the CHF + hM3Dq + CNO + EA group. **(G)** The statistical analysis of LVFS values (biological replicates *n* = 6, mean ± SD). ^***^p < 0.001 vs. the CHF + mCherry+CNO group; ^###^*p* < 0.001 vs. the CHF + mCherry+CNO + EA group; ^&&&^*p* < 0.001 vs. the CHF + hM3Dq + CNO + EA group. **(H)** The statistical analysis of NT-proBNP values (biological replicates *n* = 6, mean ± SD). ^***^p < 0.001 vs. the CHF + mCherry+CNO group; ^###^*p* < 0.001 vs. the CHF + mCherry+CNO + EA group; ^&&&^*p* < 0.001 vs. the CHF + hM3Dq + CNO + EA group. **(I)** The statistical analysis of NE values (biological replicates *n* = 6, mean ± SD). ^***^p < 0.001 vs. the CHF + mCherry+CNO group; ^###^*p* < 0.001 vs. the CHF + mCherry+CNO + EA group; ^&&&^*p* < 0.001 vs. the CHF + hM3Dq + CNO + EA group. **(J)** The statistical analysis of LF/HF ratio (biological replicates *n* = 6, mean ± SD). ^***^p < 0.001 vs. the CHF + mCherry+CNO group; ^###^*p* < 0.001 vs. the CHF + mCherry+CNO + EA group; ^&&&^*p* < 0.001 vs. the CHF + hM3Dq + CNO + EA group.

The chemogenetic strategy was employed to activate CRH neurons and assess their impact on rats with CHF undergoing EA treatment (Figures 8A,B). We verified the efficacy of chemogenetic activation. In the CHF+hM3Dq+CNO+EA group, CNO administration significantly increased the percentage of mCherry+ neurons co-expressing c-Fos compared to the CHF+hM3Dq+Saline+EA group (Figure 8C), confirming that hM3Dq-mediated activation successfully reactivated PVN neurons and counteracted the inhibitory effect of EA. The CHF+mCherry+CNO+EA group showed improved myocardial structure and reduced fibrosis compared to the CHF+mCherry+CNO group, indicating the beneficial effects of EA (Figures 8D–G). However, the CHF+hM3Dq+CNO+EA group exhibited worsened cardiac pathology, suggesting that CRH neuron activation counteracted benefits of EA. Echocardiography confirmed enhanced cardiac function in the CHF+mCherry+CNO+EA group, which was diminished in the CHF+hM3Dq+CNO+EA group. Serum NE and NT-proBNP levels were reduced in the CHF+mCherry+CNO+EA group but increased in the CHF+hM3Dq+CNO+EA group (Figures 8H,I). HRV analysis showed improved autonomic balance in the CHF+mCherry+CNO+EA group and worsened balance in the CHF+hM3Dq+CNO+EA group (Figure 8J). These results indicate that CRH neuron activation diminishes cardioprotective effects of EA in CHF rats.

## 4 Discussion

The pathogenesis of CHF following myocardial infarction is complex and multifactorial, leading not only to structural and functional abnormalities of the heart but also to potential dysregulation of the central autonomic nervous system. Recent researches have suggested that CHF may involve bidirectional interactions between the heart and the brain, particularly through the mediation of central autonomic nuclei (Mehra et al., 2022). Acupuncture, as a representative non-pharmacological therapy, has been increasingly recognized for its efficacy and safety in the treatment of CVDs (Wen et al., 2021). Moreover, the neurobiological mechanisms underlying the cardiovascular effects of acupuncture have attracted growing attention, especially its ability to modulate central autonomic circuits (Hanqing et al., 2024; Xiaotong et al., 2022). These findings provide new insights into the central mechanisms of EA-mediated cardioprotection and support its potential as a complementary strategy for CHF management.

EA is a therapeutic modality that integrates traditional acupuncture with modern electrical stimulation techniques. In our work, we revealed that EA intervention substantially improved CHF. By comparing cardiac histopathology, echocardiographic parameters, and serum levels of NE and NT-proBNP among the three groups, we observed that EA exerted a protective influence on cardiac function. Meanwhile, EA effectively reduced sympathetic hyperactivity and restored autonomic balance in the heart under CHF conditions. Modern medical research has demonstrated that EA can activate peripheral sensory nerve endings by stimulating specific acupoints, thereby transmitting signals to the central nervous system and modulating central autonomic function (Lin et al., 2022; Zhao, 2008). This peripheral-to-central neural regulatory mechanism contributes to the inhibition of excessive sympathetic activation and the improvement of cardiac autonomic imbalance (Clyburn et al., 2022; Dyavanapalli, 2020). In addition, EA can regulate neuroendocrine balance and molecular signaling pathways, thereby reducing cardiac load and improving cardiac function (Xin et al., 2022; Ma et al., 2025). At the molecular level, EA exerts its effects by modulating relevant signaling pathways to inhibit cardiomyocyte apoptosis and inflammatory responses, ultimately improving cardiac function (Li et al., 2022; Guo et al., 2021; Zuo et al., 2024). These mechanisms collectively highlight the importance of EA in ameliorating CVDs and provide a solid theoretical foundation for its application in the clinical management of these conditions.

The PVN is a key center for the integration of autonomic and neuroendocrine signals. It exerts a significant influence on cardiovascular homeostasis by regulating neuroendocrine axes and sympathetic nerve output directly related to cardiac function (Pyner, 2009; Grzęda et al., 2023). Anatomically, this regulation relies on dense projections from PVN neurons (including those expressing CRH) to the rostral ventrolateral medulla (RVLM) and the spinal intermediolateral cell column (IML; Pyner and Coote, 2000). In heart failure, hyperactivity of this PVN-RVLM/IML axis acts as a major driver of sympathetic overactivation (Kang et al., 2011). Consequently, abnormal PVN activity is closely associated with autonomic dysfunction observed in various CVDs (Iremonger and Power, 2025; Savić et al., 2022). EA involves inserting fine needles into specific acupoints and applying electrical stimulation, which strongly activates deep tissue somatic afferent fibers (Aδ and C fibers; Zhou et al., 2005). This somatosensory input is transmitted through brainstem nuclei (such as the dorsal horn and nucleus of the solitary tract) to higher central regions. Crucially, these ascending pathways project to and significantly modulate key autonomic nuclei, including the PVN, the RVLM, and the periaqueductal gray matter (Dou et al., 2023), thereby inhibiting sympathetic outflow likely via the aforementioned descending pathways. Through the integration of these ascending and descending signals, EA effectively modulates the neural circuits connecting the brainstem to the heart. This modulation restores the critical sympathetic-parasympathetic balance, not only by reducing the excessive sympathetic drive mediated by the PVN-RVLM axis, but also by enhancing parasympathetic tone (Tjen et al., 2016). It is worth noting that while the primary focus of this study was on sympathetic inhibition, the therapeutic efficacy of EA likely involves a dual mechanism that includes the simultaneous activation of the parasympathetic nervous system. Previous studies have indicated that EA can activate vagal efferent neurons in the dorsal motor nucleus of the vagus and the nucleus ambiguus, thereby enhancing vagal activity (Guo et al., 2012). This restoration of vagal tone is crucial, as it not only antagonizes sympathetic overexcitation but also exerts direct cardioprotective effects, potentially via the cholinergic anti-inflammatory pathway or by improving heart rate variability (Wu et al., 2017). Consequently, this comprehensive normalization of autonomic output from the brainstem to the heart directly alleviates adverse cardiac effects, including reduced arrhythmia incidence, improved baroreceptor reflex sensitivity, and mitigation of pathological ventricular remodeling, thereby improving overall cardiac function (Díaz et al., 2021; Toledo et al., 2017).

Moreover, the results of this study further confirm the neural connections between the heart, the HT7 acupoint, and the PVN. Our viral tracing data provides qualitative anatomical evidence supporting the existence of the Heart-PVN-HT7 axis. The functional significance of this circuit was further substantiated by our chemogenetic results. Based on the aforementioned mechanisms, we further explored the specific role of the PVN in the protective effects of EA improve CHF in a rat model of CHF. Immunofluorescence staining of the PVN in CHF model rats demonstrated a significant upregulation of c-Fos-positive neurons expression. However, EA treatment significantly attenuated this effect, further corroborating the hypothesis that the beneficial effects of EA on CHF may be mediated by the PVN. This indicates that the PVN indeed plays a significant role in the protective effects of EA against CHF in the rat model. Subsequently, *in vivo* electrophysiological results suggested that the activity of PVN neurons was enhanced in CHF rats, while EA significantly reduced this activity. Notably, we also observed that sympathetic nerve activity was enhanced in CHF model rats, a trend that was inhibited by EA. This result is consistent with the staining findings in the PVN, supporting the notion of a functional connection between the PVN and the sympathetic nervous system. Furthermore, correlation analysis revealed a significant association between PVN neuronal activity and cardiac function indices, suggesting that PVN hyperactivity tracks closely with the severity of the disease state. We also observed that the therapeutic efficacy of EA in improving CHF was diminished following lesioning of the PVN. The above findings are consistent with previous researches emphasizing the importance of PVN neuronal activity in the pathophysiology of CHF. Moreover, it highlights the crucial role of the PVN in mediating the therapeutic effects of EA on CHF.

It is well established that the sympathetic nervous system directly affects cardiac function, and its excessive activation may further exacerbate myocardial damage (Triposkiadis et al., 2024; Li, 2022). This study, through HRV analysis, revealed that rats in the EA group exhibited significantly reduced sympathetic nervous system excitability compared with the CHF group. However, given that HRV may not directly reflect sympathetic nervous system excitability, we further assessed the serum NE concentration in each group of rats. NE is an important neurotransmitter released by sympathetic nerve terminals, and changes in its concentration reflect the state of sympathetic nervous system activity. When the sympathetic nervous system is activated, NE release increases, thereby affecting cardiac function (Badrov et al., 2023). Additionally, previous researches have shown that CRH neurons play a pathological role in CHF (Zheng et al., 2012; Sivukhina et al., 2009). CRH neurons within the PVN directly innervate the RVLM and the intermediolateral cell column of the spinal cord, thereby modulating autonomic nuclei in these regions to regulate sympathetic nerve output (Shu et al., 2024). This finding lends additional support to our hypothesis that PVN^CRH^ neurons are linked to the sympathetic nervous system. To further validate this hypothesis, we conducted HRV analysis and measured NE levels in serum in experiments where PVN^CRH^ neurons were chemogenetically modulated. The results showed that inhibiting PVN^CRH^ neurons reduced sympathetic activity and significantly improved cardiac function in CHF model rats, similar to the effects of EA intervention. However, when PVN^CRH^ neurons were activated, the beneficial effects of EA intervention were offset. These results robustly corroborate our hypothesis that EA intervention ameliorates CHF by modulating the sympathetic nervous system through PVN^CRH^ neurons.

Numerous researches have shown bidirectional communication between visceral organs and the brain. In recent years, with the in-depth study of brain connectomics, increasing evidence has shown that the linkages between the body surface and viscera as well as between neurons in different brain regions are effective means of revealing the functional connectivity of brain networks (Wiegert et al., 2017; Imam et al., 2025). This also scientifically explains the specificity of acupuncture and its underlying neurobiological mechanisms. In this work, we observed that EA signals affected the activity of PVN^CRH^ neurons, which may indicate that the PVN is a crucial component in the peripheral sensory-autonomic motor regulation pathway. Moreover, the results of our study also furnish compelling evidence for the central mechanism underlying cardiac autonomic regulation. Mechanosensitive nociceptors expressed at the peripheral terminals capture the EA-induced stimulation signals, inducing peripheral sensory ganglia to generate neuroelectric signals. These signals are transmitted upward to the brain, where they are integrated, processed, and analyzed, and ultimately modulate visceral function activity via autonomic nerve output. This further corroborates our elucidation of the central mechanism underlying the improvement of CHF by EA. Furthermore, the rationale for our specific intervention protocol warrants explicit justification. The HT7 acupoint was selected based on its established efficacy in regulating cardiac function and autonomic balance in traditional Chinese medicine (Zhou et al., 2024). Importantly, the therapeutic efficacy of this specific acupoint and the stimulation protocol (2 Hz, 1 mA) has been rigorously validated in our previous studies (Wu et al., 2025; Wang W. H. et al., 2024). Regarding stimulation parameters, low-frequency EA was selected based on robust evidence demonstrating its superior sympathoinhibitory efficacy relative to high-frequency stimulation in cardiovascular disease models; this frequency-dependent mechanism has been extensively characterized in the neurophysiological literature (Zhou et al., 2005; Longhurst, 2013).

Nevertheless, this study has limitations warranting consideration. First, regarding the influence of anesthesia, EA interventions and recordings were performed under isoflurane anesthesia to minimize the confounding sympathetic surge caused by physical restraint stress in awake animals. We explicitly acknowledge that anesthesia suppresses baseline sympathetic tone and may alter HRV, neuronal firing, and c-Fos expression. However, identical anesthesia protocols were applied across all groups to ensure that the observed differences reflected the specific effects of the interventions rather than baseline shifts. Second, regarding generalizability, the exclusive inclusion of male rats and relatively small sample sizes may influence the broader applicability of our findings. Additionally, while HRV results were corroborated by serum NE levels, HRV remains an indirect measure of autonomic balance. Third, regarding mechanistic specificity, although rigorous vector controls were employed, potential off-target effects of CNO and chemogenetic DREADD tools cannot be entirely ruled out. Furthermore, we focused on PVN^CRH^ neurons without fully delineating the broader neural circuit interactions, which future studies using optogenetics should address. Finally, anatomical differences in the HT7 acupoint between rats and humans present a translational gap that requires further clinical verification.

## 5 Conclusion

In summary, our work revealed that EA exerts beneficial effects on CHF by inhibiting PVN^CRH^ neurons, with this effect being mediated through the suppression of sympathetic nerve activity. These findings elucidate the core mechanism underlying therapeutic impact of EA on CHF and lay the groundwork for its potential application in clinical practice.

## Data Availability

The original contributions presented in the study are included in the article/Supplementary material, further inquiries can be directed to the corresponding authors.

## References

[ref1] Badrov M. B. Keir D. A. Tomlinson G. Notarius C. F. Millar P. J. Kimmerly D. S. . (2023). Normal and excessive muscle sympathetic nerve activity in heart failure: implications for future trials of therapeutic autonomic modulation. Eur. J. Heart Fail. 25, 201–210. doi: 10.1002/ejhf.2749, 36459000

[ref2] Buzsáki G. (2004). Large-scale recording of neuronal ensembles. Nat. Neurosci. 7, 446–451. doi: 10.1038/nn1233, 15114356

[ref3] Chang J. W. Ramchandra R. (2025). The sympathetic nervous system in heart failure with preserved ejection fraction. Heart Fail. Rev. 30, 209–218. doi: 10.1007/s10741-024-10456-0, 39438394 PMC11646211

[ref4] Charan J. Kantharia N. D. (2013). How to calculate sample size in animal studies? J. Pharmacol. Pharmacother. 4, 303–306. doi: 10.4103/0976-500x.119726, 24250214 PMC3826013

[ref5] Chong B. Jayabaskaran J. Jauhari S. M. Chan S. P. Goh R. Kueh . (2024). Global burden of cardiovascular diseases: projections from 2025 to 2050. Eur. J. Prev. Cardiol. 32, 1001–1015. doi: 10.1093/eurjpc/zwae281 39270739

[ref6] Clyburn C. Sepe J. J. Habecker B. A. (2022). What gets on the nerves of cardiac patients? Pathophysiological changes in cardiac innervation. J. Physiol. 600, 451–461. doi: 10.1113/jp281118, 34921407 PMC8810748

[ref7] Cui S. Wang K. Wu S. B. Zhu G. Q. Cao J. Zhou Y. P. . (2018). Electroacupuncture modulates the activity of the hippocampus-nucleus tractus solitarius-vagus nerve pathway to reduce myocardial ischemic injury. Neural Regen. Res. 13, 1609–1618. doi: 10.4103/1673-5374.237124, 30127122 PMC6126117

[ref8] Dampney R. A. Michelini L. C. Li D. P. Pan H. L. (2018). Regulation of sympathetic vasomotor activity by the hypothalamic paraventricular nucleus in normotensive and hypertensive states. Am. J. Physiol. Heart Circ. Physiol. 315, H1200–H1214. doi: 10.1152/ajpheart.00216.2018, 30095973 PMC6297824

[ref9] Díaz H. S. Andrade D. C. Toledo C. Schwarz K. G. Pereyra K. V. Díaz-Jara E. (2021). Inhibition of brainstem endoplasmic reticulum stress rescues cardiorespiratory dysfunction in high output heart failure. Hypertension 77, 718–728. doi: 10.1161/hypertensionaha.120.16056, 33307852

[ref10] Dou Z. Su N. Zhou Z. Mi A. Xu L. Zhou J. (2023). Modulation of visceral pain by brain nuclei and brain circuits and the role of acupuncture: a narrative review. Front. Neurosci. 17:1243232. doi: 10.3389/fnins.2023.1243232, 38027491 PMC10646320

[ref11] Dyavanapalli J. (2020). Novel approaches to restore parasympathetic activity to the heart in cardiorespiratory diseases. Am. J. Physiol. Heart Circ. Physiol. 319, H1153–H1161. doi: 10.1152/ajpheart.00398.2020, 33035444 PMC7792713

[ref12] Fan H. Yang J. W. Wang L. Q. Huang J. Lin L. L. Wang Y. (2020). The hypotensive role of acupuncture in hypertension: clinical study and mechanistic study. Front. Aging Neurosci. 12:138. doi: 10.3389/fnagi.2020.00138, 32523527 PMC7261879

[ref13] Goldberger J. J. Arora R. Buckley U. Shivkumar K. (2019). Autonomic nervous system dysfunction: JACC focus seminar. J. Am. Coll. Cardiol. 73, 1189–1206. doi: 10.1016/j.jacc.2018.12.064, 30871703 PMC6958998

[ref14] Grzęda E. Ziarniak K. Sliwowska J. H. (2023). The paraventricular nucleus of the hypothalamus - the concertmaster of autonomic control. Focus on blood pressure regulation. Acta Neurobiol. Exp. (Wars) 83, 34–44. doi: 10.55782/ane-2023-004, 37078812

[ref15] Guo H. H. Jing X. Y. Chen H. Xu H. X. Zhu B. M. (2021). STAT3 but not STAT5 contributes to the protective effect of electroacupuncture against myocardial ischemia/reperfusion injury in mice. Front Med (Lausanne). 8:649654. doi: 10.3389/fmed.2021.649654, 34307396 PMC8299366

[ref16] Guo Z. L. Li M. Longhurst J. C. (2012). Nucleus ambiguus cholinergic neurons activated by acupuncture: relation to enkephalin. Brain Res. 1442, 25–35. doi: 10.1016/j.brainres.2012.01.006, 22306033 PMC3288561

[ref17] Guo Z. Ni H. Lu Y. Cui Z. Wang Y. Zhu Z. (2026). TNEA regulates hippocampal oscillation by improving inhibitory synaptic plasticity to ameliorates cognitive impairment in Alzheimer's disease. Adv Sci (Weinh). 13:e10885. doi: 10.1002/advs.202510885, 41215663 PMC12806385

[ref18] Hanqing X. I. Xia L. I. Ziyi Z. Xiang C. Xianghong J. Bing Z. . (2024). Neuro- and immuno-modulation mediated by the cardiac sympathetic nerve: a novel insight into the anti-ischemic efficacy of acupuncture. J. Tradit. Chin. Med. 44, 1058–1066. doi: 10.19852/j.cnki.jtcm.20240423.001, 39380238 PMC11462539

[ref19] He B. Lu Z. He W. Huang B. Jiang H. (2016). Autonomic modulation by electrical stimulation of the parasympathetic nervous system: an emerging intervention for cardiovascular diseases. Cardiovasc. Ther. 34, 167–171. doi: 10.1111/1755-5922.12179, 26914959

[ref20] Herman J. P. Mcklveen J. M. Ghosal S. Kopp B. Wulsin A. Makinson R. . (2016). Regulation of the hypothalamic-pituitary-adrenocortical stress response. Compr. Physiol. 6, 603–621. doi: 10.1002/cphy.c150015 27065163 PMC4867107

[ref21] Imam F. T. Gillespie T. H. Ziogas I. Surles-Zeigler M. C. Tappan S. Ozyurt B. I. (2025). Developing a multiscale neural connectivity knowledgebase of the autonomic nervous system. Front. Neuroinform. 19:1541184. doi: 10.3389/fninf.2025.1541184, 40162160 PMC11949889

[ref22] Iremonger K. J. Power E. M. (2025). The paraventricular nucleus of the hypothalamus: a key node in the control of behavioural states. J. Physiol. 603, 2231–2243. doi: 10.1113/jp288366, 40119815 PMC12013795

[ref23] Jiang Z. Rajamanickam S. Justice N. J. (2018). Local corticotropin-releasing factor signaling in the hypothalamic paraventricular nucleus. J. Neurosci. 38, 1874–1890. doi: 10.1523/jneurosci.1492-17.2017, 29352046 PMC5824736

[ref24] Kang Y. M. Zhang A. Q. Zhao X. F. Cardinale J. P. Elks C. (2011). Paraventricular nucleus corticotrophin releasing hormone contributes to sympathoexcitation via interaction with neurotransmitters in heart failure. Basic Res. Cardiol. 106, 473–483. doi: 10.1007/s00395-011-0155-2 21287352 PMC3118407

[ref25] Kun W. Jie Z. Shuai C. Xin W. U. Guoqi Z. Shengbing W. U. (2023). Electroacupuncture ameliorates cardiac dysfunction in myocardial ischemia model rats: a potential role of the hypothalamic-pituitary-adrenal axis. J. Tradit. Chin. Med. 43, 944–954. doi: 10.19852/j.cnki.jtcm.20230727.001, 37679982 PMC10465846

[ref26] Li Y. L. (2022). Stellate ganglia and cardiac sympathetic overactivation in heart failure. Int. J. Mol. Sci. 23:13311. doi: 10.3390/ijms232113311, 36362099 PMC9653702

[ref27] Li D. P. Pan H. L. (2007). Glutamatergic inputs in the hypothalamic paraventricular nucleus maintain sympathetic vasomotor tone in hypertension. Hypertension 49, 916–925. doi: 10.1161/01.Hyp.0000259666.99449.74, 17309953

[ref28] Li X. Wang L. Ying X. Zheng Y. Tan Q. Yu X. (2022). Electroacupuncture pre-treatment alleviates sepsis-induced cardiac inflammation and dysfunction by inhibiting the calpain-2/STAT3 pathway. Front. Physiol. 13:961909. doi: 10.3389/fphys.2022.961909, 36160853 PMC9489935

[ref29] Lin J. G. Kotha P. Chen Y. H. (2022). Understandings of acupuncture application and mechanisms. Am. J. Transl. Res. 14, 1469–1481. 35422904 PMC8991130

[ref30] Litwin S. E. Katz S. E. Morgan J. P. Douglas P. S. (1994). Serial echocardiographic assessment of left ventricular geometry and function after large myocardial infarction in the rat. Circulation 89, 345–354. 8281668 10.1161/01.cir.89.1.345

[ref31] Liu L. Tang Y. Baxter G. D. Yin H. Tumilty S. (2021). Complementary and alternative medicine - practice, attitudes, and knowledge among healthcare professionals in New Zealand: an integrative review. BMC Complement Med Ther. 21:63. doi: 10.1186/s12906-021-03235-z, 33583417 PMC7882070

[ref32] Longhurst J. (2013). Acupuncture's cardiovascular actions: a mechanistic perspective. Med Acupunct. 25, 101–113. doi: 10.1089/acu.2013.0960, 24761168 PMC3616410

[ref33] Luther J. A. Tasker J. G. (2000). Voltage-gated currents distinguish parvocellular from magnocellular neurones in the rat hypothalamic paraventricular nucleus. J. Physiol. 523, 193–209. 10673555 10.1111/j.1469-7793.2000.t01-1-00193.x PMC2269788

[ref34] Ma J. Yin X. Cui K. Wang J. Li W. Xu S. (2025). Mechanisms of acupuncture in treating depression: a review. Chin. Med. 20:29. doi: 10.1186/s13020-025-01080-7, 40033393 PMC11877828

[ref35] Manolis A. A. Manolis T. A. Apostolopoulos E. J. Apostolaki N. E. Melita H. Manolis A. S. (2021). The role of the autonomic nervous system in cardiac arrhythmias: the neuro-cardiac axis, more foe than friend? Trends Cardiovasc. Med. 31, 290–302. doi: 10.1016/j.tcm.2020.04.011, 32434043

[ref36] Mark A. L. (1995). Sympathetic dysregulation in heart failure: mechanisms and therapy. Clin. Cardiol. 18, I3–I8. 7743696 10.1002/clc.4960181303

[ref37] Mehra R. Tjurmina O. A. Ajijola O. A. Arora R. Bolser D. C. Chapleau M. W. (2022). Research opportunities in autonomic neural mechanisms of cardiopulmonary regulation: a report from the National Heart, Lung, and Blood Institute and the National Institutes of Health Office of the director workshop. JACC Basic Transl Sci. 7, 265–293. doi: 10.1016/j.jacbts.2021.11.003, 35411324 PMC8993767

[ref38] Nunn N. Womack M. Dart C. Barrett-Jolley R. (2011). Function and pharmacology of spinally-projecting sympathetic pre-autonomic neurones in the paraventricular nucleus of the hypothalamus. Curr. Neuropharmacol. 9, 262–277. doi: 10.2174/157015911795596531, 22131936 PMC3131718

[ref39] Pfeffer M. A. Pfeffer J. M. Fishbein M. C. Fletcher P. J. Spadaro J. Kloner R. A. . (1979). Myocardial infarct size and ventricular function in rats. Circ. Res. 44, 503–512. 428047 10.1161/01.res.44.4.503

[ref40] Pyner S. (2009). Neurochemistry of the paraventricular nucleus of the hypothalamus: implications for cardiovascular regulation. J. Chem. Neuroanat. 38, 197–208. doi: 10.1016/j.jchemneu.2009.03.005, 19778682

[ref41] Pyner S. (2014). The paraventricular nucleus and heart failure. Exp. Physiol. 99, 332–339. doi: 10.1113/expphysiol.2013.072678, 24317407

[ref42] Pyner S. Coote J. H. (2000). Identification of branching paraventricular neurons of the hypothalamus that project to the rostroventrolateral medulla and spinal cord. Neuroscience 100, 549–556. 11098118 10.1016/s0306-4522(00)00283-9

[ref43] Quiroga R. Q. Nadasdy Z. Ben-Shaul Y. (2004). Unsupervised spike detection and sorting with wavelets and superparamagnetic clustering. Neural Comput. 16, 1661–1687. doi: 10.1162/089976604774201631, 15228749

[ref44] Ran J. Zhou P. Wang J. Zhao X. Huang Y. Zhou Q. (2025). Global, regional, and national burden of heart failure and its underlying causes, 1990-2021: results from the global burden of disease study 2021. Biomark. Res. 13:16. doi: 10.1186/s40364-025-00728-8, 39849627 PMC11755835

[ref45] Savić B. Murphy D. Japundžić-Žigon N. (2022). The paraventricular nucleus of the hypothalamus in control of blood pressure and blood pressure variability. Front. Physiol. 13:858941. doi: 10.3389/fphys.2022.858941, 35370790 PMC8966844

[ref46] Shao Y. Li Y. Wang B. Li C. Chen H. (2025). The value of electroacupuncture in the treatment of coronary heart disease: a review of the mechanisms and clinical studies of electroacupuncture therapy. Cardiol. Res. Pract. 2025:4684871. doi: 10.1155/crp/4684871, 40654523 PMC12255498

[ref47] Shu Q. Zhou J. Zhang B. Zhang F. Zhou X. Wu Y. (2024). Electroacupuncture alleviates myocardial ischemia-reperfusion injury by inhibiting hypothalamic paraventricular nucleus neurons projecting to the rostral ventrolateral medulla. Eur. J. Neurosci. 60, 4861–4876. doi: 10.1111/ejn.16480, 39054660

[ref48] Sivukhina E. V. Poskrebysheva A. S. Smurova Iu V. Dolzhikov A. A. Morozov Iu E. Jirikowski G. F. . (2009). Altered hypothalamic-pituitary-adrenal axis activity in patients with chronic heart failure. Horm. Metab. Res. 41, 778–784. doi: 10.1055/s-0029-1224182 19544245

[ref49] Tjen A. L. S. C. Guo Z. L. Fu L. W. Longhurst J. C. (2016). Paraventricular nucleus modulates excitatory cardiovascular reflexes during electroacupuncture. Sci. Rep. 6:25910. doi: 10.1038/srep25910, 27181844 PMC4867624

[ref50] Toledo C. Andrade D. C. Lucero C. Arce-Alvarez A. Díaz H. S. Aliaga V. (2017). Cardiac diastolic and autonomic dysfunction are aggravated by central chemoreflex activation in heart failure with preserved ejection fraction rats. J. Physiol. 595, 2479–2495. doi: 10.1113/jp273558, 28181258 PMC5390883

[ref51] Triposkiadis F. Briasoulis A. Kitai T. Magouliotis D. Athanasiou T. Skoularigis J. (2024). The sympathetic nervous system in heart failure revisited. Heart Fail. Rev. 29, 355–365. doi: 10.1007/s10741-023-10345-y, 37707755

[ref52] Triposkiadis F. Karayannis G. Giamouzis G. Skoularigis J. Louridas G. Butler J. (2009). The sympathetic nervous system in heart failure physiology, pathophysiology, and clinical implications. J. Am. Coll. Cardiol. 54, 1747–1762. doi: 10.1016/j.jacc.2009.05.015, 19874988

[ref53] Wan F. Cao Y. Wang Q. Y. Yang J. W. Wang L. Liu C. Z. (2025). Electroacupuncture improves cerebral blood flow in vascular cognitive impairment mice by activating the locus coeruleus - prefrontal cortex circuit. Neuroscience 583, 33–42. doi: 10.1016/j.neuroscience.2025.07.026, 40683522

[ref54] Wang S. Fang R. Huang L. Zhou L. Liu H. Cai M. (2024). Acupuncture in traditional chinese medicine: a complementary approach for cardiovascular health. J. Multidiscip. Healthc. 17, 3459–3473. doi: 10.2147/jmdh.S476319, 39050695 PMC11268752

[ref55] Wang L. Sheng G. Cui J. Yao Y. Bai X. Chen F. (2022). Electroacupuncture attenuates ischemic injury after stroke and promotes angiogenesis via activation of EPO mediated Src and VEGF signaling pathways. PLoS One 17:e0274620. doi: 10.1371/journal.pone.0274620, 36108080 PMC9477374

[ref56] Wang W. H. Zeng Q.-L. Zhang J.-J. Wu H.-S. Wu S.-B. Zhou M.-Q. (2024). Electroacupuncture improves myocardial fibrosis in heart failure rats by attenuating ECM collagen deposition through modulation of TGF-β1/Smads signaling pathway. Traditional Med. Res. 9, 43–48. doi: 10.53388/TMR20240118001

[ref57] Wen J. Chen X. Yang Y. Liu J. Li E. Liu J. . (2021). Acupuncture medical therapy and its underlying mechanisms: a systematic review. Am. J. Chin. Med. 49, 1–23. doi: 10.1142/s0192415x21500014, 33371816

[ref58] Wiegert J. S. Mahn M. Prigge M. Printz Y. Yizhar O. (2017). Silencing neurons: tools, applications, and experimental constraints. Neuron 95, 504–529. doi: 10.1016/j.neuron.2017.06.050, 28772120 PMC5830081

[ref59] Wu S. J. Li Y. C. Shi Z. W. Lin Z. H. Rao Z. H. Tai S. C. . (2017). Alteration of cholinergic anti-inflammatory pathway in rat with ischemic cardiomyopathy-modified electrophysiological function of heart. J. Am. Heart Assoc. 6:e006510. doi: 10.1161/jaha.117.006510, 28928157 PMC5634297

[ref60] Wu H. S. Zhu L. Liu P. Liu X. H. Su H. Zheng X. Y. (2025). Neural mechanism of HT7 electroacupuncture in myocardial ischemia: critical role of the paraventricular nucleus oxytocin system. Front. Neurosci. 19:1678938. doi: 10.3389/fnins.2025.1678938, 41141422 PMC12546331

[ref61] Xiaotong W. Liaoyuan L. I. Yating Z. Qi S. Shuaiya W. Pianpian C. (2022). Electroacupuncture preconditioning alleviates myocardial ischemia-reperfusion injury through the hypothalamic paraventricular nucleus- interposed nucleus nerve pathway. J. Tradit. Chin. Med. 42, 379–388. doi: 10.19852/j.cnki.jtcm.2022.03.005, 35610007 PMC9924790

[ref62] Xin Y. Y. Wang J. X. Xu A. J. (2022). Electroacupuncture ameliorates neuroinflammation in animal models. Acupunct. Med. 40, 474–483. doi: 10.1177/09645284221076515, 35229660

[ref63] Xu R. Zheng K. Pan Y. Li P. Chen M. Nie H. (2026). Electroacupuncture ameliorates incisional pain via suppressing IL-33 signaling-related macrophage infiltration and ROS overproduction in incised skin. Chin. Med. 21:26. doi: 10.1186/s13020-025-01273-0, 41514473 PMC12784544

[ref64] Yang C. Li X. Hu M. Li T. Jiang L. Zhang Y. (2024). Gut microbiota as predictive biomarker for chronic heart failure in patients with different nutritional risk. J. Cardiovasc. Transl. Res. 17, 1240–1257. doi: 10.1007/s12265-024-10529-3, 38913293

[ref65] Yuan G. Han A. Wu J. Lu Y. Zhang D. Sun Y. (2018). Bao Yuan decoction and Tao Hong Si Wu decoction improve lung structural remodeling in a rat model of myocardial infarction: possible involvement of suppression of inflammation and fibrosis and regulation of the TGF-β1/Smad3 and NF-κB pathways. Biosci. Trends 12, 491–501. doi: 10.5582/bst.2018.01242, 30473557

[ref66] Zhao Z. Q. (2008). Neural mechanism underlying acupuncture analgesia. Prog. Neurobiol. 85, 355–375. doi: 10.1016/j.pneurobio.2008.05.004, 18582529

[ref67] Zheng M. Kang Y. M. Liu W. Zang W. J. Bao C. Y. Qin D. N. (2012). Inhibition of cyclooxygenase-2 reduces hypothalamic excitation in rats with adriamycin-induced heart failure. PLoS One 7:e48771. doi: 10.1371/journal.pone.0048771, 23152801 PMC3496718

[ref68] Zhou W. Fu L. W. Tjen A. L. S. C. Li P. Longhurst J. C. (2005). Afferent mechanisms underlying stimulation modality-related modulation of acupuncture-related cardiovascular responses. J. Appl. Physiol. (1985) 98, 872–880. doi: 10.1152/japplphysiol.01079.2004, 15531558

[ref69] Zhou X. Yang P. Dong C. Chang H. Zhang F. Shu Q. (2025). Electroacupuncture pretreatment alleviates myocardial ischemia-reperfusion injury by inhibiting engulfment by microglia in the lateral hypothalamus. CNS Neurosci. Ther. 31:e70595. doi: 10.1111/cns.70595, 40904234 PMC12409074

[ref70] Zhou J. Zhang B. Zhou X. Zhang F. Shu Q. Wu Y. . (2024). Electroacupuncture pretreatment mediates sympathetic nerves to alleviate myocardial ischemia-reperfusion injury via CRH neurons in the paraventricular nucleus of the hypothalamus. Chin. Med. 19:43. doi: 10.1186/s13020-024-00916-y, 38448912 PMC10916233

[ref71] Zipes D. P. (1990). Influence of myocardial ischemia and infarction on autonomic innervation of heart. Circulation 82, 1095–1105. 2205413 10.1161/01.cir.82.4.1095

[ref72] Zuo H. Qu Q. Tong Y. Wang L. Wang X. Wu S. (2024). Electroacupuncture alleviates acute myocardial ischemic injury in mice by regulating the β(1) adrenergic receptor and post-receptor protein kinase a signaling pathway. Acupunct. Med. 42, 342–355. doi: 10.1177/09645284241298716, 39579035 PMC11633077

