# Supplementary material for: Electroacupuncture ameliorates chronic heart failure: the role of CRH neurons in the paraventricularnucleus of the hypothalamus
Source: Front Neurosci. 2026 Feb 25;20:1741523. doi: 10.3389/fnins.2026.1741523 (PMC12975747; doi:10.3389/fnins.2026.1741523)
Supplement: Supplementary file 1 [file Table_1.DOCX]

**Table S1.** Summary of animal grouping and experimental design

| Experiment | Group Name | n | Model | PVN Intervention (Stereotaxic Injection) | Treatment  (IP Injection / EA) |
| --- | --- | --- | --- | --- | --- |
| Exp. I  (Basics) | Sham | 6 | Sham | / | / |
|  | CHF | 6 | CHF | / | / |
|  | EA | 6 | CHF | / | EA |
|  | Sham EA | 6 | CHF | / | Sham EA |
|  | Tracing Sub-study | 9 (Each 3) | Intact | Heart / HT7 / PVN (Tracer/CTB) |  |
| Exp. II  (Lesion) | CHF+Saline | 6 | CHF | Saline | / |
|  | CHF+Saline+EA | 6 | CHF | Saline | EA |
|  | CHF+KA | 6 | CHF | Kainic Acid (Lesion) | / |
|  | CHF+KA+EA | 6 | CHF | Kainic Acid (Lesion) | EA |
| Exp. III  (Inhibition) | Sham+mCherry+CNO | 6 | Sham | AAV-mCherry (Control) | CNO |
|  | CHF+mCherry+CNO | 6 | CHF | AAV-mCherry (Control) | CNO |
|  | CHF+hM4Di+CNO | 6 | CHF | AAV-hM4Di (Inhibitory) | CNO |
|  | CHF+hM4Di+Saline | 6 | CHF | AAV-hM4Di (Inhibitory) | Saline |
| Exp. IV  (Excitation) | CHF+mCherry+CNO | 6 | CHF | AAV-mCherry (Control) | CNO |
|  | CHF+mCherry+CNO+EA | 6 | CHF | AAV-mCherry (Control) | CNO+EA |
|  | CHF+hM3Dq+CNO+EA | 6 | CHF | AAV-hM3Dq (Excitatory) | CNO+EA |
|  | CHF+hM3Dq+Saline+EA | 6 | CHF | AAV-hM3Dq (Excitatory) | Saline + EA |

CHF: chronic heart failure; EA: electroacupuncture; PVN: paraventricular nucleus; KA: kainic acid; CNO: clozapine-N-oxide; IP: intraperitoneal
